# Vaginal and neonatal microbiota in pregnant women with preterm premature rupture of membranes and consecutive early onset neonatal sepsis

**DOI:** 10.1186/s12916-023-02805-x

**Published:** 2023-03-13

**Authors:** Luiz Gustavo dos Anjos Borges, Jana Pastuschek, Yvonne Heimann, Kristin Dawczynski, PEONS study group, Michael Bergner, Roland Haase, Johannes Stubert, Dirk Olbertz, Iris Plumeier, Silke Kahl, Ann Kathrin Heroven, Ekkehard Schleußner, Dietmar H. Pieper, Janine Zöllkau

**Affiliations:** 1 Microbial Interactions and Processes Research Group, Helmholtz Centre for Infection Research, Inhoffenstrasse 7, 38124 Brunswick, Germany; 2 Department of Obstetrics, Jena University Hospital, Am Klinikum 1, 07747 Jena, Germany; 3 Center for Sepsis Control and Case (CSCC), Jena University Hospital, Am Klinikum 1, 07747 Jena, Germany; 4 Department of Pediatrics, Section Neonatology, Jena University Hospital, Am Klinikum 1, 07747 Jena, Germany

**Keywords:** Vaginal microbiota, Neonatal microbiota, Meconium, Preterm premature rupture of the membranes, Early-onset neonatal sepsis

## Abstract

**Background:**

Preterm premature rupture of membranes (PPROM), which is associated with vaginal dysbiosis, is responsible for up to one-third of all preterm births. Consecutive ascending colonization, infection, and inflammation may lead to relevant neonatal morbidity including early-onset neonatal sepsis (EONS). The present study aims to assess the vaginal microbial composition of PPROM patients and its development under standard antibiotic therapy and to evaluate the usefulness of the vaginal microbiota for the prediction of EONS. It moreover aims to decipher neonatal microbiota at birth as possible mirror of the in utero microbiota.

**Methods:**

As part of the PEONS prospective multicenter cohort study, 78 women with PPROM and their 89 neonates were recruited. Maternal vaginal and neonatal pharyngeal, rectal, umbilical cord blood, and meconium microbiota were analyzed by 16S rRNA gene sequencing. Significant differences between the sample groups were evaluated using permutational multivariate analysis of variance and differently distributed taxa by the Mann–Whitney test. Potential biomarkers for the prediction of EONS were analyzed using the MetaboAnalyst platform.

**Results:**

Vaginal microbiota at admission after PPROM were dominated by *Lactobacillus* spp. Standard antibiotic treatment triggers significant changes in microbial community (relative depletion of *Lactobacillus* spp. and relative enrichment of *Ureaplasma parvum*) accompanied by an increase in bacterial diversity, evenness and richness. The neonatal microbiota showed a heterogeneous microbial composition where meconium samples were characterized by specific taxa enriched in this niche. The vaginal microbiota at birth was shown to have the potential to predict EONS with *Escherichia/Shigella* and *Facklamia* as risk taxa and *Anaerococcus obesiensis* and *Campylobacter ureolyticus* as protective taxa. EONS cases could also be predicted at a reasonable rate from neonatal meconium communities with the protective taxa *Bifidobacterium longum*, *Agathobacter rectale*, and *S. epidermidis* as features.

**Conclusions:**

Vaginal and neonatal microbiota analysis by 16S rRNA gene sequencing after PPROM may form the basis of individualized risk assessment for consecutive EONS. Further studies on extended cohorts are necessary to evaluate how far this technique may in future close a diagnostic gap to optimize and personalize the clinical management of PPROM patients.

**Trial registration:**

NCT03819192, ClinicalTrials.gov. Registered on January 28, 2019.

**Supplementary Information:**

The online version contains supplementary material available at 10.1186/s12916-023-02805-x.

## Background

According to the WHO (https://www.who.int/news-room/fact-sheets/detail/preterm-birth), 15 million babies are born preterm (before 37 completed weeks of gestation) every year, and preterm birth complications are the leading cause of death among children under 5 years of age, accounting for approximately 1 million deaths in 2015 [1]. In 30 to 40% of the cases, a preterm premature rupture of membranes (PPROM) is preceding the preterm deliveries [2]. Such a rupture of the membranes provides an entry point for ascending microbes to the uterine cavity which may contribute to the development of chorioamnionitis, colonization of the neonate, and subsequent maternal and neonatal morbidities [3, 4].

Neonatal sepsis is a frequently fatal condition affecting neonates and children worldwide [5, 6]. Based on the time of infection, neonatal sepsis is classified as early-onset neonatal sepsis (EONS), occurring in the first 3 days of life and being caused by bacterial pathogens transmitted vertically from mother to infant before or during delivery [5, 6] whereas late-onset neonatal sepsis (LONS) is sepsis occurring after 72 h, which may be caused by vertically or horizontally acquired pathogens. The incidence of EONS in a healthy term birth pregnancy is lower than one case per 1000 births [7], whereas the rate of developing EONS after PPROM ranges from 14 to 22% [4, 8].

The importance of the vaginal microbiome for the prevention of urogenital diseases in women and for the maintenance of health has been more and more realized [9]. In healthy women, the vaginal microbiota is typically dominated by different *Lactobacillus* species. By producing lactic acid, *Lactobacilli* create a low pH in the vaginal environment, which inhibit growth of potential pathogens [10]. However, microbiota composition and stability differ between women and are mainly dependent on the menstrual cycle phases, sexual activity, and ethnicity [11]. A dramatic depletion of *Lactobacillus* spp. is considered a vaginal dysbiosis and is characterized by an enhanced growth of pathogenic organisms such as *Gardnerella*, *Prevotella*, *Atopobium*, or *Fannyhessea* [12, 13]. Vaginal dysbiosis has been associated with several pathological conditions such as bacterial vaginosis, increased risk for sexually transmitted infections, preterm labor, preterm premature rupture of membrane, or chorioamnionitis [4, 14, 15]. *Lactobacillus* spp. stability in pregnancy was suggested to represent an evolutionary adaptation to enhance reproductive fitness and protect against ascending infections [16].

The clinical management of PPROM is challenging and has to balance the prolongation of the pregnancy to enable fetal maturation and the risk of infection and subsequent poor neonatal outcomes. In most countries, and specifically when PPROM occurs at earlier gestational ages (< 34 weeks), antibiotic therapy is recommended during pregnancy prolongation to reduce neonatal morbidity [17, 18]. However, a recent analysis indicated that erythromycin treatment resulted in a shift towards dysbiotic community structures and depletion of *Lactobacillus* spp. [3, 4] thereby disrupting optimal communities. It was thus suggested that it is time to reconsider the role of antibiotic therapy in PPROM [3].

Several perinatal factors like maternal nutrition, antibiotic use, and maternal stress, as well as maternal age during pregnancy, gestational age, mode of delivery, and breastfeeding, are described frequently to modulate the acquisition and development of gut microbiota in early life [19]. However, it was generally assumed that the neonate is born sterile and only after delivery populated by bacteria. In contrast, in the recent years, various reports indicated the presence of bacteria or at least of bacterial DNA in the placenta, uterus, and amniotic fluid [20, 21]. With a rupture of the membranes, the barrier to the fetus is damaged which makes bacterial contamination of the amniotic fluid and finally of the fetus feasible. In fact, 25% of preterm infants are reported to be born to a mother with intra-amniotic infection, most commonly due to invasion of the amniotic cavity by *Ureaplasma* species [22]. Placental pathogenic colonization seeded from ascending vaginal infection [16] and the post-birth meconium microbiome and other fetal microbiota might therefore mirror the in utero microbial environment [23].

The present study aims to assess the vaginal microbial composition of PPROM patients and its development under standard antibiotic therapy and to evaluate the usefulness of the vaginal microbiota for the prediction of EONS. It moreover aims to decipher neonatal microbiota at birth as a possible mirror of the in-utero microbiota in the context of EONS after PPROM.

## Methods

### Study population

This study is part of the PEONS trial (NCT03819192, ClinicalTrials.gov), a prospective multicenter case-cohort study for follow-up of pregnant women with preterm premature rupture of membranes (PPROM) with hospitalization between 22^+0^ and 34^+0^ weeks of gestation and their respective neonates. PPROM was detected clinically during the gynecological examination and, if necessary, by using biochemical amniotic fluid point-of-care testing (e.g., AmniSure or ROMPlus test). A total of 78 women and their 89 neonates (11 twin pregnancies) were included in the study. All 78 patients received antibiotic therapy after diagnosis of PPROM according to national guidelines and the study center standard. The guideline-compliant initial calculated antibiotic therapy was performed with aminopenicillin in all 78 patients and supplemented with a macrolide in the majority of cases. Depending on the clinical course, adjustment, discontinuation, or continuation was made at the discretion of the treating physician (Additional file 1: Table S1). The childbirth of most PPROM patients was by Cesarean Sect. (57.7%). Further 41% of neonates were delivered by spontaneous vaginal birth and two cases by assisted delivery with vacuum extraction (Additional file 2: Table S2). Decision on neonatal antibiotic therapy was made by the treating neonatologist depending on the clinical presentation, the standard of the treating clinic, and the latest microbiological diagnostics and anti-infective treatment of the mother. Forty-five neonates underwent antibiotic treatment (18 EONS, 27 non-EONS). Antibiotics contained mainly ampicillin (36 neonates) and gentamicin (36 neonates). In a few cases, tazobatam (8 neonates), cefotaxime (6 neonates), meropenem (14 neonates), teicoplanin (3 neonates), vancomycin (12 neonates), clarithromycin (13 neonates), erythromycin (2 neonates), metronidazole (1 neonate), ceftazidim (2 neonates), or cefuroxim (1 neonate) were used.

### Sample collection

Swabs for microbiome analysis were collected longitudinally of the pregnant women up to delivery and within the first 2 days of life of the neonates. Maternal vaginal samples were collected by sterile FLOQSwabs (Copan) at up to three time points from every pregnant woman with PPROM. An initial sample (V0) was collected at the first clinical examination after confirmation of PPROM and prior to antibiotic therapy. A second sample was aimed to be collected after 2–6 days of antibiotic treatment (V1), when available. V1 samples were therefore not available if delivery occurred prior to 2 days of antibiotic administration. The last maternal sampling was performed within 24 h before delivery (V2). The V2 samples were sub-grouped based on the antibiotic treatment duration before delivery, as the antibiotic treatment had a significant effect on the vaginal community structure only when administrated for > 48 h (see also results section). All samples taken < 48 h after the start of antibiotic treatment were considered to be similar in microbial community structure to those samples collected at the hospital admission. These samples collected < 48 h antibiotic treatment (V2e samples, V2_early_) were thus considered equivalent to V0 samples, and subject to be used as a V0 sample when the true one was absent. All samples taken between > 48 and < 170 h of antibiotic treatment were considered as equivalent to V1 samples. The V2 samples having received antibiotics for > 48 h and < 170 h were termed V2m (V2_medium_). V2 samples of patients taken after > 170 h of antibiotic treatment were termed V2l (V2_late_, a subset of V2m), and patients where AB treatment was terminated > 48 h before delivery were termed V2r (V2r_recovered_, a subset of V2l) (Additional file 3: Table S3).

Neonatal samples were collected from 4 different body sites of newborn infants. Umbilical cord blood (UC), rectal swabs (RE), and pharyngeal swabs (PH) were collected at the delivery using sterile FLOQSwabs (Copan), while the meconium (ME) sample was collected prior to 48 h of life. Eighty-four neonates were successfully sampled to evaluate the microbiome at the first moment of life. The amounts of 79 pharyngeal swabs, 81 rectal swabs, 74 blood samples, and 75 meconium samples were submitted to the laboratory.

All swabs and meconium samples collected for the microbiome analysis were immediately frozen at − 80 °C (freezing up to 24 h at − 20 °C was suitable).

### DNA extraction and sequencing

DNA was extracted using the FastDNA™SpinKit for Soil (MP Biomedicals, USA), according to the manufacturer’s instruction. Briefly, it comprises mechanical lysis using a Fast Prep®-24 (MP Biomedicals, USA). A 3-step PCR approach was used to amplify the V1V2 variable regions of the 16S rRNA gene. PCR with primers 27Bif (3′-AGRGTTHGATYMTGGCTCAG-5′) optimized to reliably amplify also *Bifidobacteriaceae* and 338R (5′-TGCTGCCTCCCGTAGGAGT-3′) for 20 cycles was used to enrich for target sequences followed by a second PCR with 15 cycles using the same primers with a short overhang and a third amplification step of 10 cycles that added the two indices and Illumina adapters to amplicons [24]. Amplified products were purified, normalized, and pooled using the SequalPrep Normalization Plate and subjected to 2 × 300-bp paired-end Illumina MiSeq sequencing (Illumina, San Diego, CA, USA). To control for contaminations possibly originating from chemicals and extraction kits used, PBS samples were extracted and processed in the same way on a regular basis.

### Data processing

The fastQ files were analyzed with the dada2 package version 1.12.1 in R [25]. The quality trimming and filtering steps were performed using the filterAndTrim function. Forward and reverse reads were trimmed on the 5′-end by 20 and 19 bases, respectively. Reads were truncated to a length of 240 bases, and a maximum of 2 expected errors per read was permitted. After denoising and paired-end reads merging, chimeras were removed. Sequence variants were annotated based on the naïve Bayesian classification with a pseudo-bootstrap threshold of 80% using RDP set18 [26]. The remaining non-bacterial sequences (eukaryota, mitochondria, chloroplast) were manually deleted. Sequence variants were then manually analyzed against the RDP database using the Seqmatch function to define the discriminatory power of each sequence type. Species-level annotations were assigned to a sequence variant when only 16S rRNA gene fragments of previously described isolates of a single species were aligned with a maximum of two mismatches with this sequence variant [27] (Additional file 4: Table S4).

The analysis of extraction control samples revealed the presence of slight but varying contamination levels in the FastDNA™SpinKit for Soil, stably comprising *Burkholderia* and/or *Ralstonia* sequence types (Additional file 5: Table S5), which may disturb the analysis in low-biomass neonatal samples. Correlation analysis was performed to identify these potential contaminants. Spearman correlations were calculated for sequence variants with relative abundances > 0.1% and a prevalence > 10% in neonatal samples and a network constructed based on pairwise Spearman correlations (*p* > 0.4) using the psych package version v1.9.12.31 in R [28]. The network was visualized using Cytoscape (version 3.7.2) and analyzed. Sequence types forming a major cluster dominated by *Ralstonia solanacearum* (ST2), and *Burkholderia caribensis* (ST34) and identified as major contaminants originating from the extraction kit used, were removed before downstream analysis. Sequence types of a lower mean abundance (> 0.1%) but belonging to the same species as those removed, as well as Planctomycetia (ST25) and Acidobacteria (ST46) forming a small cluster and exhibiting at least 2 strong correlations (*p* > 0.4) were removed. All sequence variants removed have also been indicated previously as originating from contaminants arising from different sources [29, 30]. After cleaning, samples with less than 2000 reads were removed from the analysis, as they comprised mainly reads originating from contaminations and thus only negligible amounts of target bacterial DNA (Additional file 6: Table S6).

### Statistical analysis

Absolute abundance counts were rarefied using the *rarecurve* function from the vegan R package [31]. Rarefied data were used to determine the alpha diversity indices. The diversity, richness, and evenness were estimated by Shannon’s index, Chao1’s index, and the Pielou index, respectively. Indices were determined using the Microbiome R package [32], and significant differences between the groups were determined by the Mann–Whitney test.

The data matrices comprising relative abundances of sequence variants, species, and genera were used to construct sample-similarity matrices using the Bray–Curtis (BC) algorithm which were visualized using non-metric multidimensional scaling (nMDS). Heatmaps were plotted using the pheatmap package [33]. Significant differences between a priori predefined groups were evaluated using permutational multivariate analysis of variance (PERMANOVA). Groups of samples were considered significantly different if the *p*-value was < 0.05. Differently distributed species-level taxa were determined by the Kruskal–Wallis test or the Mann–Whitney test. Differences in the presence/absence of specific taxa were evaluated using Fisher’s exact test. The Benjamini–Hochberg correction was applied for multiple comparisons [34]. Only taxa that were present in the community of at least 10% of the samples were considered. Groups of samples were considered significantly different if the adjusted *p*-value (*q*) was < 0.05. If the uncorrected *p*-value was < 0.05, groups of samples were assumed to trend to be different. Dunn’s test was used as a post hoc test following the Kruskal–Wallis test, and the corrected test was performed when multiple tests had been applied.

Multivariate analyses were performed using PRIMER (V.7.0.11, PRIMER-E, Plymouth Marine Laboratory, UK) and univariate analyses using Prism 8 (GraphPad Software).

Potential biomarkers were analyzed using the MetaboAnalyst 5.0 analysis platform (https://www.metaboanalyst.ca/MetaboAnalyst/) [35] using cube root-transformed species abundance data.

## Results

### Composition of the vaginal microbiota of PPROM patients

The 16S rRNA gene sequencing was performed for 166 vaginal swab samples collected at hospital admission (V0), after antibiotic treatment (V1), and < 24 h before delivery (V2). The overall upstream analyses resulted in 2923 amplicon sequence variants (ASVs) assigned to 20 different phyla and 752 species (Additional file 7: Table S7). Almost half of the vaginal ASVs (1401/2983 ASVs) were observed in V0 samples (*n* = 78), belonging to 13 phyla and 483 species.

**Fig. 1:**
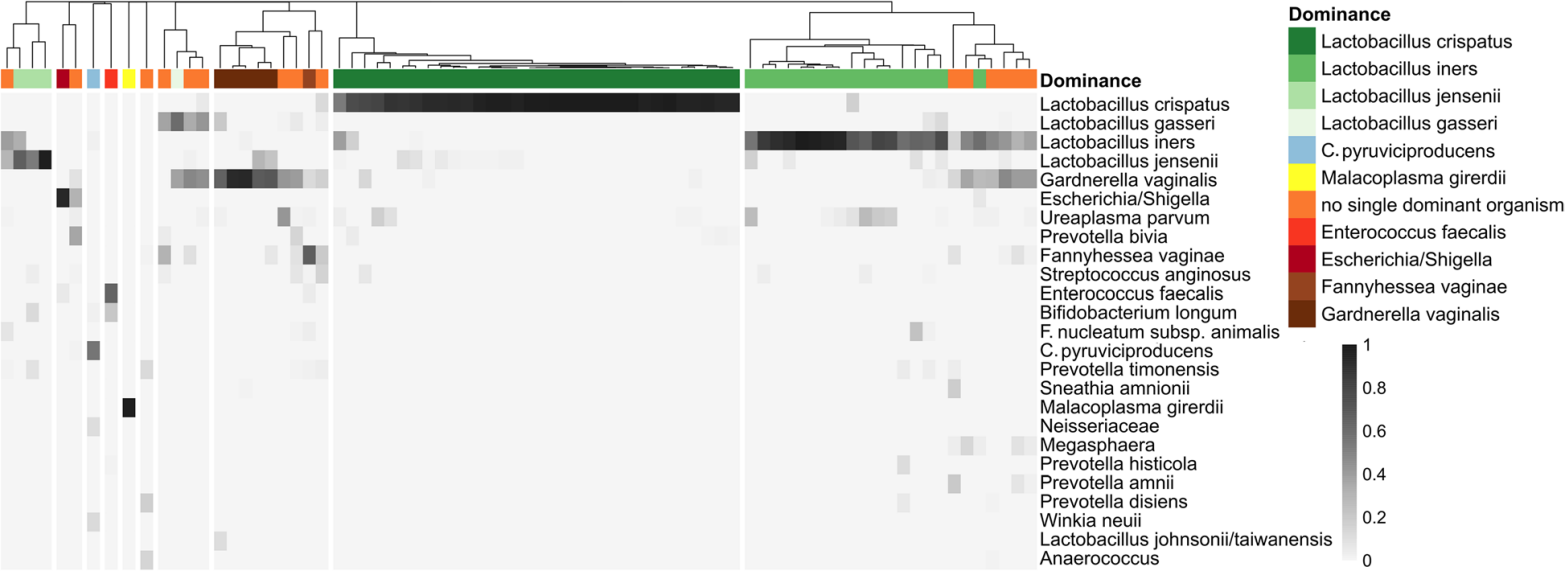
Microbial composition of vaginal samples of PPROM patients at hospital admission (V0). Heatmap includes taxa with a relative abundance > 10% in at least one sample. Samples where a single taxon shows a relative abundance > 50% of relative abundance are indicated by a specific color code (dominance group). Hierarchical cluster based on Bray–Curtis similarity analysis of vaginal bacterial communities of V0 samples (*n* = 78)

At the time of the hospital admission (V0), 65.4% of the pregnant women harbored a vaginal microbiota dominated by *Lactobacillus* species (rel. abundance > 50%), mainly *L. crispatus* (32/78) and *L. iners* (17/78). *Lactobacillus jensenii* (3/78) and *L. gasseri* (1/78) were also dominant in a few samples (Fig. 1). This is in accordance with the data of previous reports comparing the vaginal microbiota in PPROM patients with those of controls, where PPROM patients were less often colonized by a *Lactobacillus* dominated community compared to controls (56–77% compared to 81–91%) [36, 37].

These communities dominated by *Lactobacillus* sp. have been termed community states I, III, V, and II [9]. They were characterized by a low diversity as indicated by a Shannon index (*H*′) below 1.5 (often < 1). Besides *Lactobacillus* sp., *Gardnerella vaginalis* was highly abundant and dominant in five samples. In another five samples, *G. vaginalis* and *L. iners* were equally abundant and together accumulated > 70% of sequence reads. Similarly, *G. vaginalis* often co-occurred with *L. gasseri*, and in four samples, they together comprised > 70% of sequence reads. In contrast, the presence of a high abundance of *L. crispatus* seems to be incompatible with a high abundance of *G. vaginalis*. Other species only seldomly become dominating. *Malacoplasma girerdii*, *Corynebacterium pyruviciproducens*, *Enterococcus faecalis*, *Fannyhessea vaginae*, and *Escherichia*/*Shigella* were dominating in one sample each, with only the sample dominated by *Malacoplasma girerdii* exhibiting an extremely low diversity (*H*′ = 0.24).

### Composition of the vaginal microbiota during antibiotic treatment

Comparison of microbial community composition based on their Bray–Curtis similarities showed that out of 23 samples taken < 2 days after starting of antibiotic administration only 4 exhibited a similarity < 50% to the community present before the antibiotic treatment. This indicates that in most of these cases, the antibiotic treatment time was insufficient to change the bacterial community composition on the 16S rDNA level. In contrast, the microbial community remained relatively stable (> 50% similarity) in only 4 out of those 40 cases where antibiotic was administrated for > 2 days (Additional file 8: Fig. S1).

**Fig. 2:**
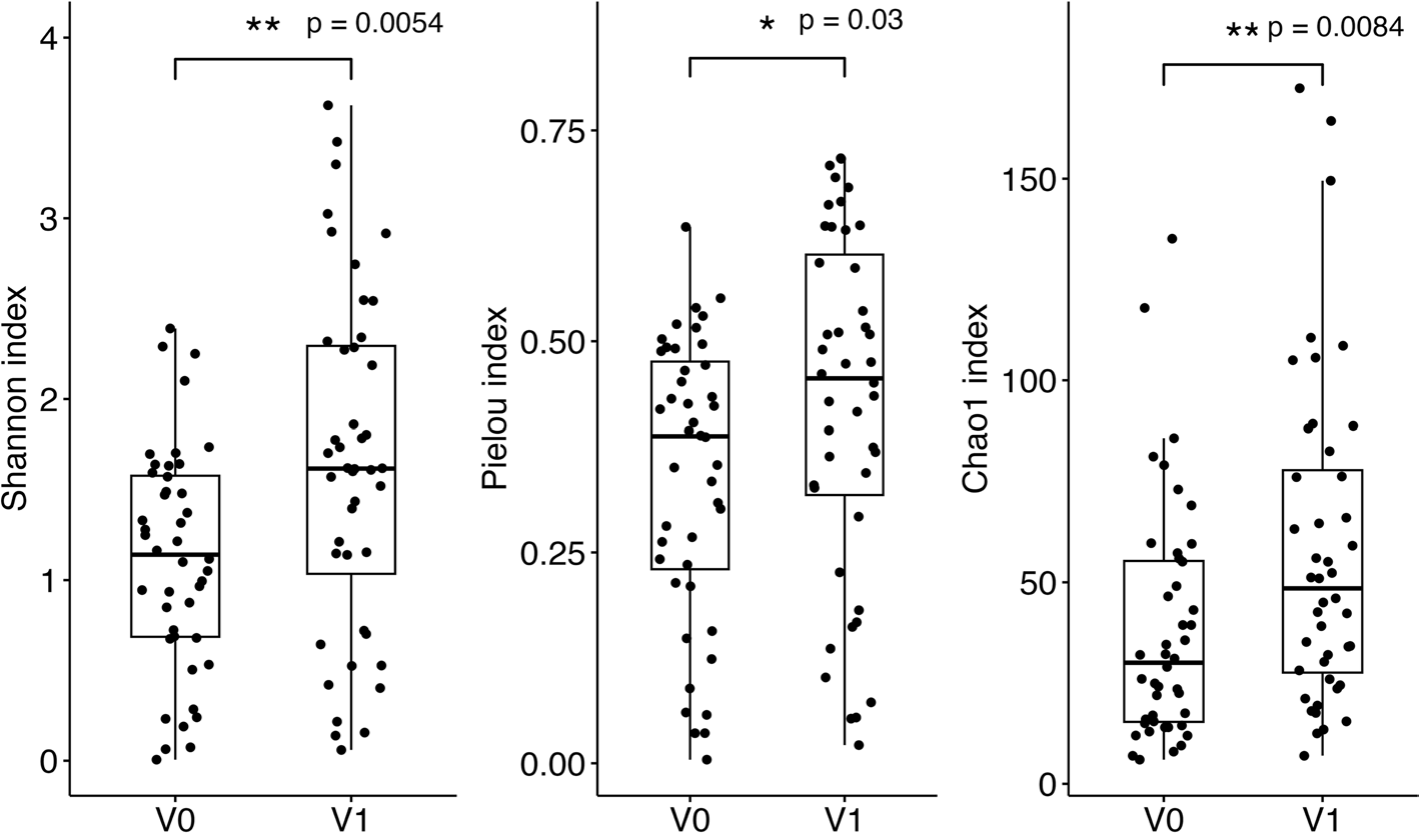
Vaginal community diversity indicated by Shannon diversity (*H*′), Pielou’s evenness (*J′*), and Chao1 richness. Statistically significant differences between the groups of samples (V0 and V1) are indicated by **p < 0.01 and *p < 0.05. Quartiles and median are shown

Then, alpha-diversity indices of 44 pairs of V0 and V1 samples were analyzed (samples being collected at hospital admission or < 2 days thereafter versus samples of patients having been treated with antibiotics longer than 2 days). A significant increase in bacterial diversity (*H*′; *p* = 0.0054) was observed (Fig. 2). The increase in bacterial diversity was accompanied by an increase in evenness (Pielou index; *p* = 0.03) and richness (Chao1 index; *p* = 0.0084).

**Fig. 3:**
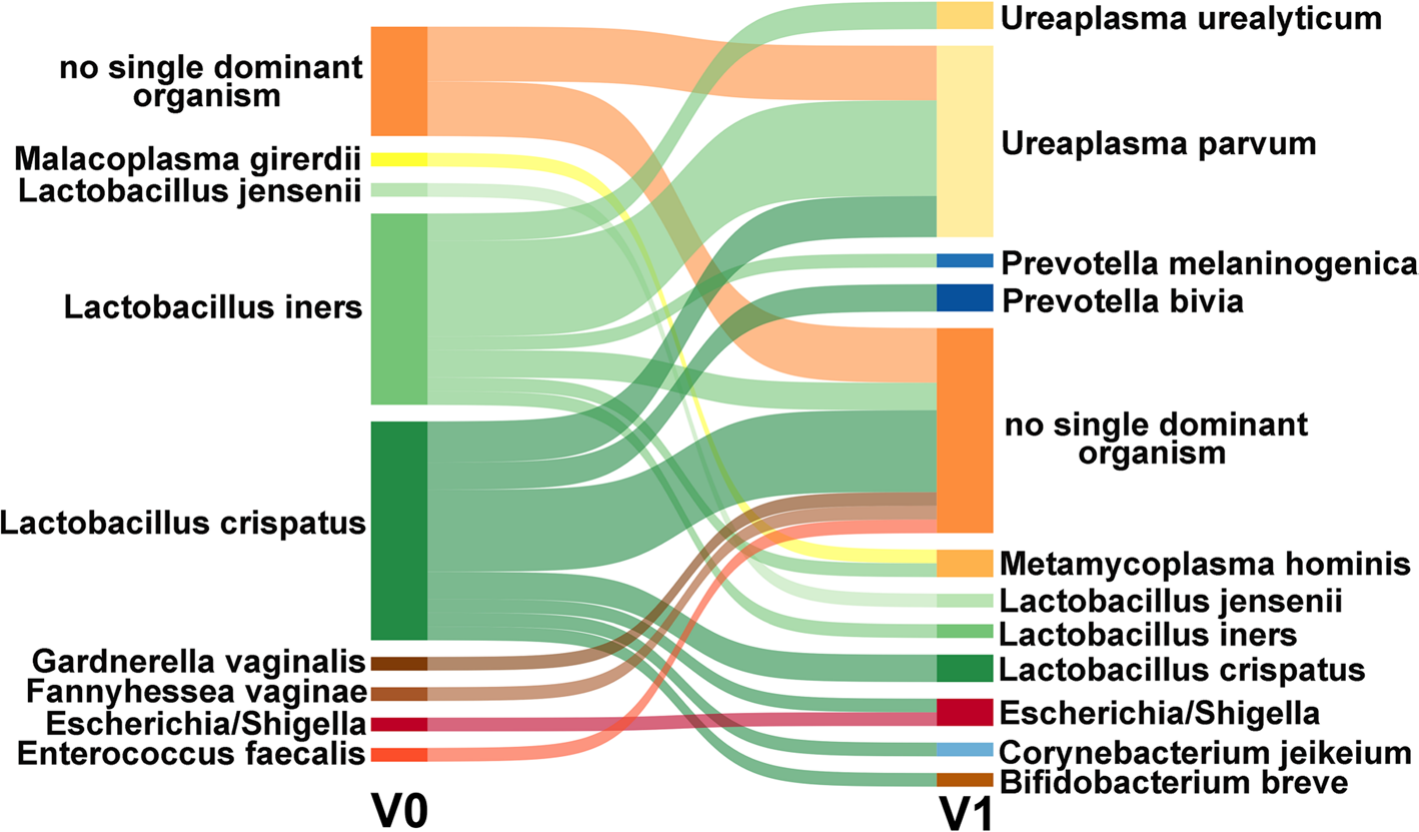
Changes in the dominant bacterial taxon of vaginal samples during antibiotic treatment. A taxon is defined as dominant if it is present in a relative abundance > 50%. Only samples of those 44 patients are shown, where both samples during hospital admission (V0) and samples taken between > 48 and < 170 h of antibiotic treatment (V1) were available

Thirty-one out of these 44 patients had a vaginal microbiota dominated by *Lactobacillus species* before antibiotic treatment (Fig. 1). As reported, antibiotic treatment had a tremendous effect on *Lactobacillus* spp. abundance in most of these samples (Fig. 3), and similarities between V0 and V1 communities were below 50%. However, in five patients (J01, R11, R18, H15—*Lactobacillus* species; J18—*Escherichia/Shigella*), the community remained similar.

**Fig. 4:**
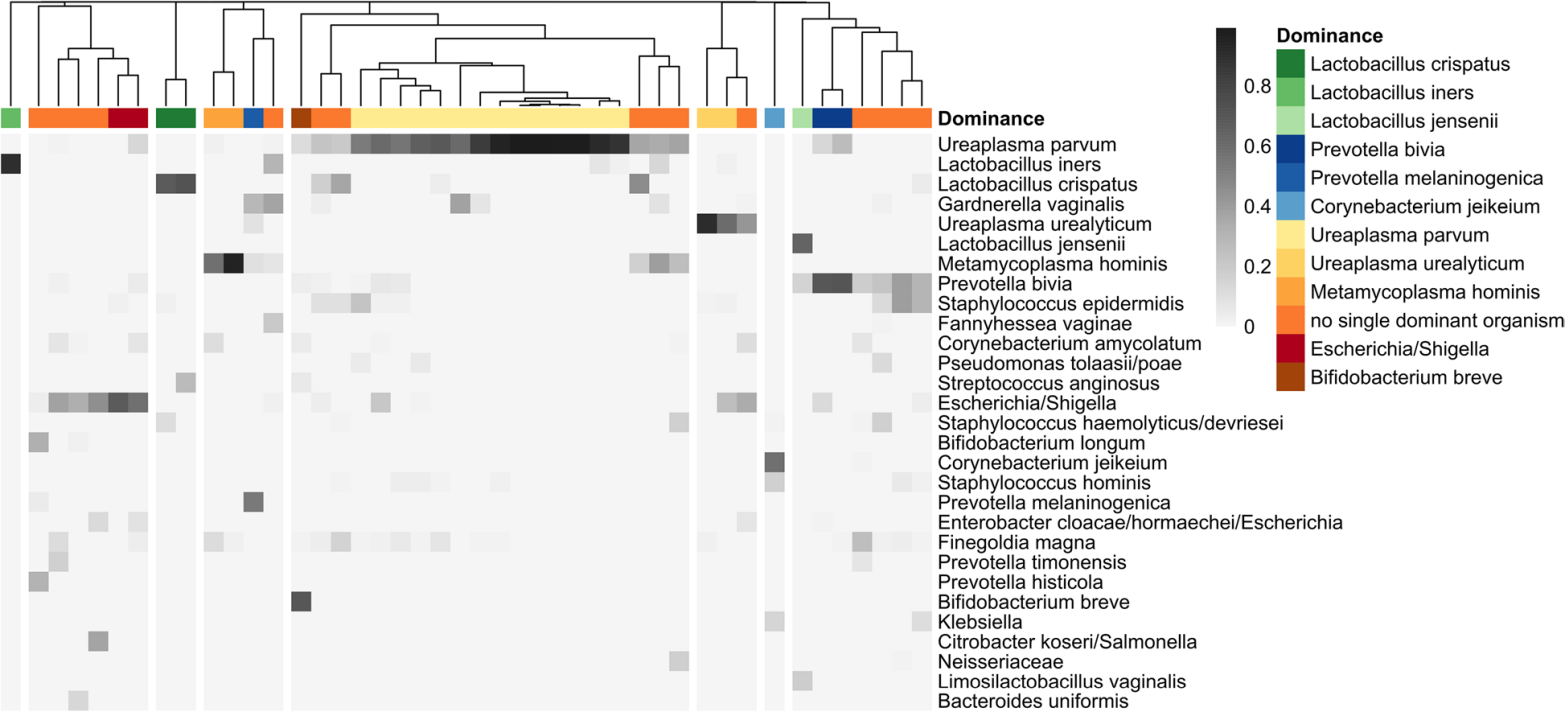
Microbial composition of vaginal samples of PPROM patients during antibiotic therapy (V1). Heatmap includes taxa with a relative abundance > 10% in at least one sample. Samples where a single taxon shows a relative abundance > 50% of relative abundance are indicated by a specific color code (dominance group). Hierarchical cluster based on Bray–Curtis dissimilarity analysis of vaginal bacterial communities of V1 samples (*n* = 44)

The microbiota analysis showed 14 V1 samples being dominated by *Ureaplasma parvum* (Figs. 3 and 4), with 8 of them exhibiting a low diversity (*H*′ < 1). Although *U. parvum* was frequently observed in V0 samples, it was not dominant in any vaginal microbiota before antibiotic treatment (Figs. 1 and 3). A few cases were dominated by *Bifidobacterium breve*, *Corynebacterium jeikeium*, *Escherichia/Shigella*, *Metamycoplasma hominis*, *Prevotella bivia*, *P. melaninogenica*, or *U. urealyticum*.

### Composition of vaginal microbiota at the time of delivery

A total of 63 samples were collected right before delivery. Twenty-nine of these V2 samples were collected < 2 days after admission to the hospital (V2e). They typically were similar to the respective V0 samples (> 50%) and only 4 V2e had a community composition considerably different from that observed at hospital admission (J07, J35, R04, and R13; < 33% similarity). In accordance, twelve V2e samples were dominated by *L. crispatus* (12/29; 41%) and another six samples by *L. iners*, *L. jensenii*, or *L. gasseri* (6/29; 21%) (Additional file 9: Fig. S2).

Thirty-four V2 samples were collected after the patient had received antibiotics for at least 2 days. For 22 of those patients, a sampling set of V0 (at admission), V1 (between 2 and 6 days of antibiotic treatment), and V2l (after extended antibiotic treatment) was available (Additional file 10: Fig. S3). The microbial composition remained stable in 4 patients at V1 (R11, R18, J01, and J18, V0–V1 similarity > 50%), even though the extended antibiotic treatment resulted in the eradication of *Lactobacillus* spp. from those communities. The only community that remained stable (J18, V0–V1, and V0–V2 similarity > 50%) was dominated by *Escherichia/Shigella* already at hospital admission.

Interestingly, V2l samples of patients that received antibiotics until the time of birth did not change dramatically in microbial composition (43.9 ± 19.5% similarity) upon extended antibiotic treatment (between V1 and V2l samples), and 6 of 10 samples (H11, H18, J29, J46, R03, and R09) kept a similarity > 50% (Additional file 10: Fig. S3). In contrast, the microbial composition changed more dramatically (V1–V2, similarity 24.0 ± 22.7%; *p* = 0.042) in patients that stopped the antibiotic treatment at least 2 days before delivery (V2r) (Additional file 10: Fig. S3). However, in only one case (J42), the microbial composition recovered to that observed before the antibiotic treatment, and V2r became dominated by *L. crispatus* (Additional file 9: Fig. S2).

### Composition of neonatal microbiota

**Fig. 5:**
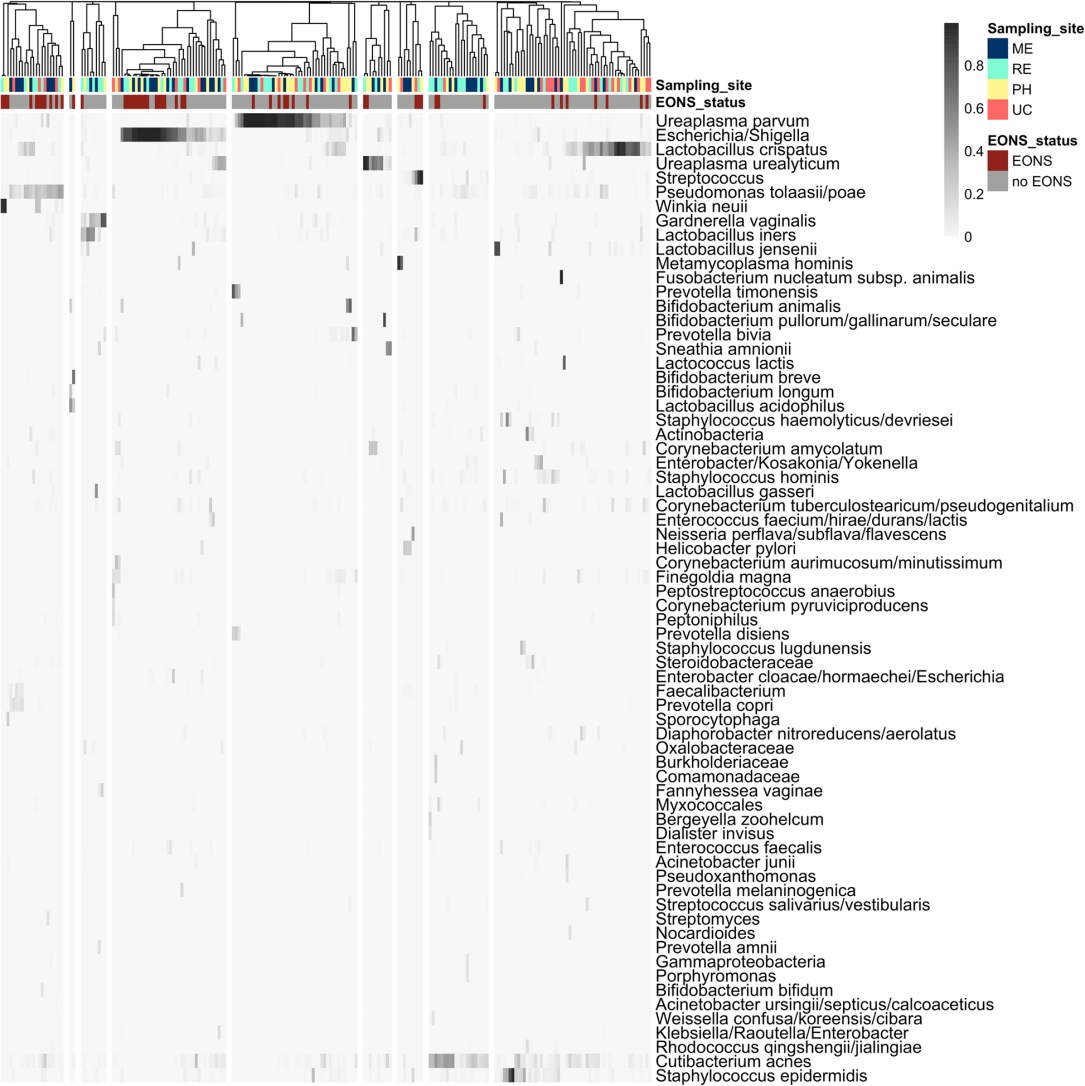
Microbial composition of neonatal samples. Heatmap includes taxa with a relative abundance > 10% in at least one sample. Hierarchical clusters are based on the Bray–Curtis dissimilarity analysis of bacterial composition. The heatmap includes meconium samples (ME, *n* = 60), rectal swab samples (RE, *n* = 58), pharyngeal swab samples (PH, *n* = 55), and umbilical cord blood samples (UC, *n* = 39)

Samples collected from 84 neonates were processed for sequencing. Ninety-seven of 309 samples (31.4%) did not yield an amplification product or a sufficient number of qualified reads. A total of 60 meconium, 58 rectal swabs, 55 pharyngeal swabs, and 39 umbilical cord blood samples passed through the quality control and were analyzed (Additional file 11: Table S8). Overall, the neonatal microbiota showed a heterogeneous microbial composition with multiple species in high relative abundance (Fig. 5).

**Fig. 6:**
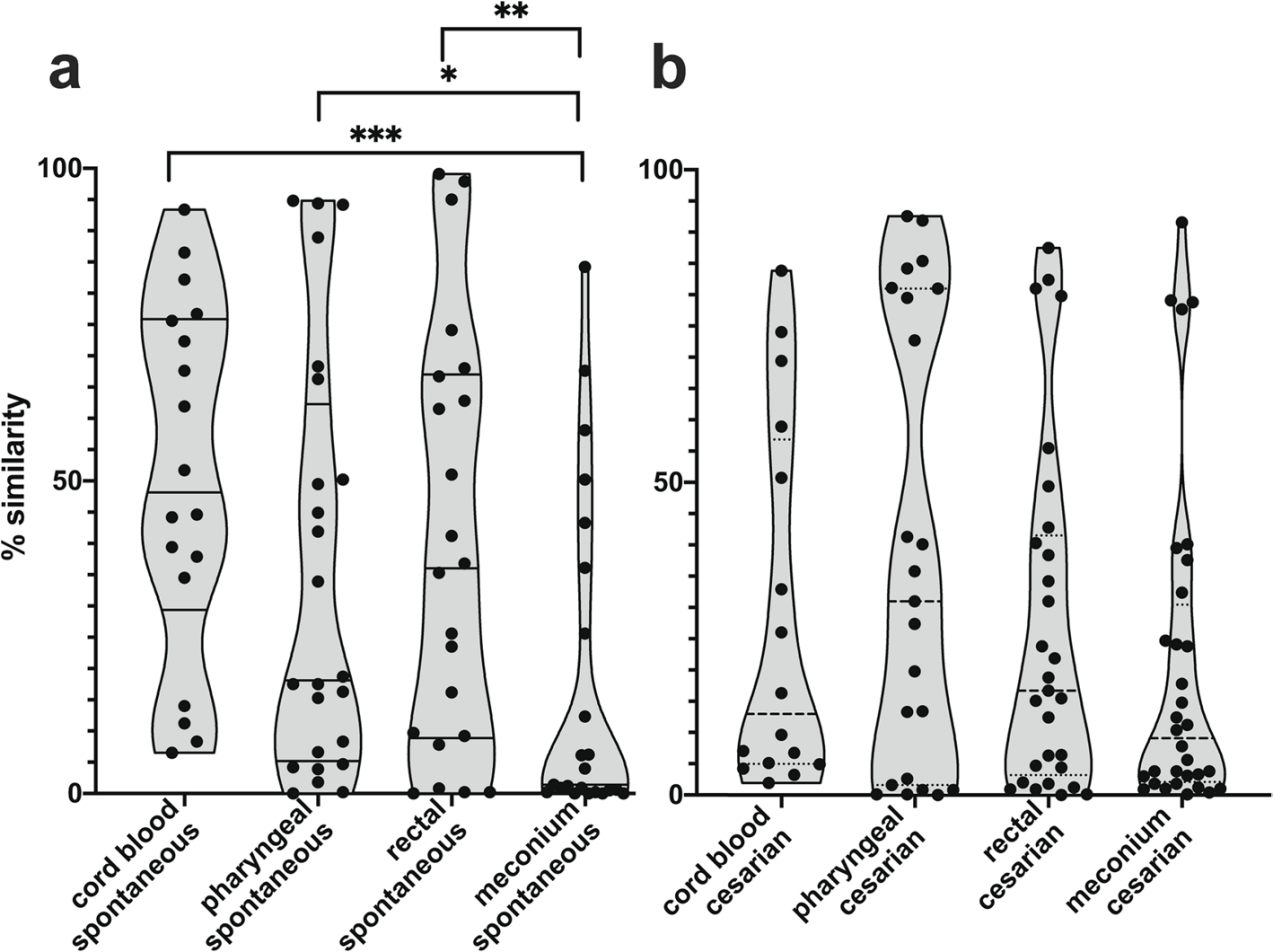
Similarities in microbial community structure between the vaginal microbiota at the time of birth and neonatal microbiota. The Bray–Curtis similarities are given based on standardized species abundance data. **a** Similarity between V2 samples and umbilical cord blood (*n* = 18), pharyngeal swabs (*n* = 24), rectal swabs (*n* = 22), and meconium samples (*n* = 23) of neonates delivered by spontaneous birth. **b** Similarity between V2 samples and umbilical cord blood (*n* = 16), pharyngeal swabs (*n* = 23), rectal swabs (*n* = 29), and meconium samples (*n* = 32) of neonates delivered by C-section. The difference in similarity to V2 samples between neonatal communities of different origins was analyzed by the Kruskal–Wallis test, and a significant difference in pairwise comparisons is indicated with *p < 0.05, **p < 0.01, and ***p < 0.001

A comparison of the Bray–Curtis similarities between the vaginal microbiota at the time of birth and the respective neonatal microbiota showed that, specifically, the umbilical cord blood samples of neonates born by vaginal delivery had a community composition highly similar to those of the mothers. This indicates that the microbial community on umbilical cord blood samples are remnants of the maternal microbiota from the umbilical cord surface rather than the microbial content of the neonatal bloodstream. Accordingly, bacterial communities determined from the umbilical cord blood of neonates born by Cesarean section were significantly less similar to those of the mother (*p* = 0.0184) (Fig. 6). In contrast, meconium samples from neonates born by vaginal delivery had a lower similarity to the V2 microbial community compared to other neonatal sites sampled. Therefore, meconium microbiota has to be considered as minimally contaminated in dependency of birth mode and as a reliable microbiota source representing a possible intrauterine bacterial colonization after PPROM.

Whereas the vaginal microbiota of early delivery mothers (V2e, < 2 days antibiotic treatment) and late delivery mothers (V2m > 2 days antibiotic treatment) were in fact significantly different (Table 1), there was no significant difference in the meconium communities of the respective children indicating that antibiotics given to the mothers do not exert a significant effect on the meconium communities.

**Table 1:** Pairwise Results for PERMANOVA based on the Bray–Curtis similarities of vaginal and meconium communities calculated from standardized relative species abundance data of early (< 48 h) and late (> 48 h) delivery mothers and their neonates

Pairwise comparison	Pseudo-*F*	*p*
V2m, MEm	1.7867	0.001*
V2e, MEe	2.0372	0.001*
V2m, V2e	2.0353	0.001*
Mem, MEe	0.8829	0.740

*V2m*, vaginal communities of mothers with delivery after > 2 days of antibiotic treatment; *MEm*, meconium communities of mothers with delivery after > 2 days of antibiotic treatment; *V2e*, vaginal communities of mothers with delivery after < 2 days of antibiotic treatment; *MEe*, meconium communities of mothers with delivery after < 2 days of antibiotic treatment

^*^Significant value

However, even though vaginal and meconium communities were significantly different and a significant effect of antibiotic treatment of the mother was not observed on meconium communities, some correlations between vaginal and meconium samples were obvious, such as the dominance of *L. crispatus* in the meconium samples of neonate H21, or even, the dominance of *U. parvum* and *Escherichia/Shigella* in meconium samples of neonates J13 and J18, respectively (Additional file 12: Fig. S4). Besides maternal vaginal microbiota, skin inhabitants such as *Cutibacterium acnes* and typical gastrointestinal organisms, such as *Helicobacter pylori*, were observed in meconium samples (Additional file 12: Fig. S4).

We observed above that communities characterized from umbilical cord blood samples exhibited some similarities with maternal vaginal samples. To analyze if there were or were no significant differences between umbilical cord blood samples as well as pharyngeal and rectal swab communities, these were compared to those of the mothers at the time of delivery. The 2-way PERMANOVA (Additional file 13: Table S9) revealed that V2 communities differed from the corresponding pharyngeal, rectal, and umbilical cord blood samples. However, there was no statistically significant difference between the neonatal samples. This holds both for early delivery and late delivery samples. As observed for meconium communities, a significant difference between early and late delivery communities was only evident for vaginal but not for any neonatal communities.

To analyze in more detail the differences between the bacterial communities, the difference in the distribution of taxa was compared between vaginal (V2) and neonatal samples (ME, PH, RE, NB). Clearly, V2 was the most distinct, and various bacterial species and genera were differentially distributed (Additional file 14: Table S10).

**Fig. 7:**
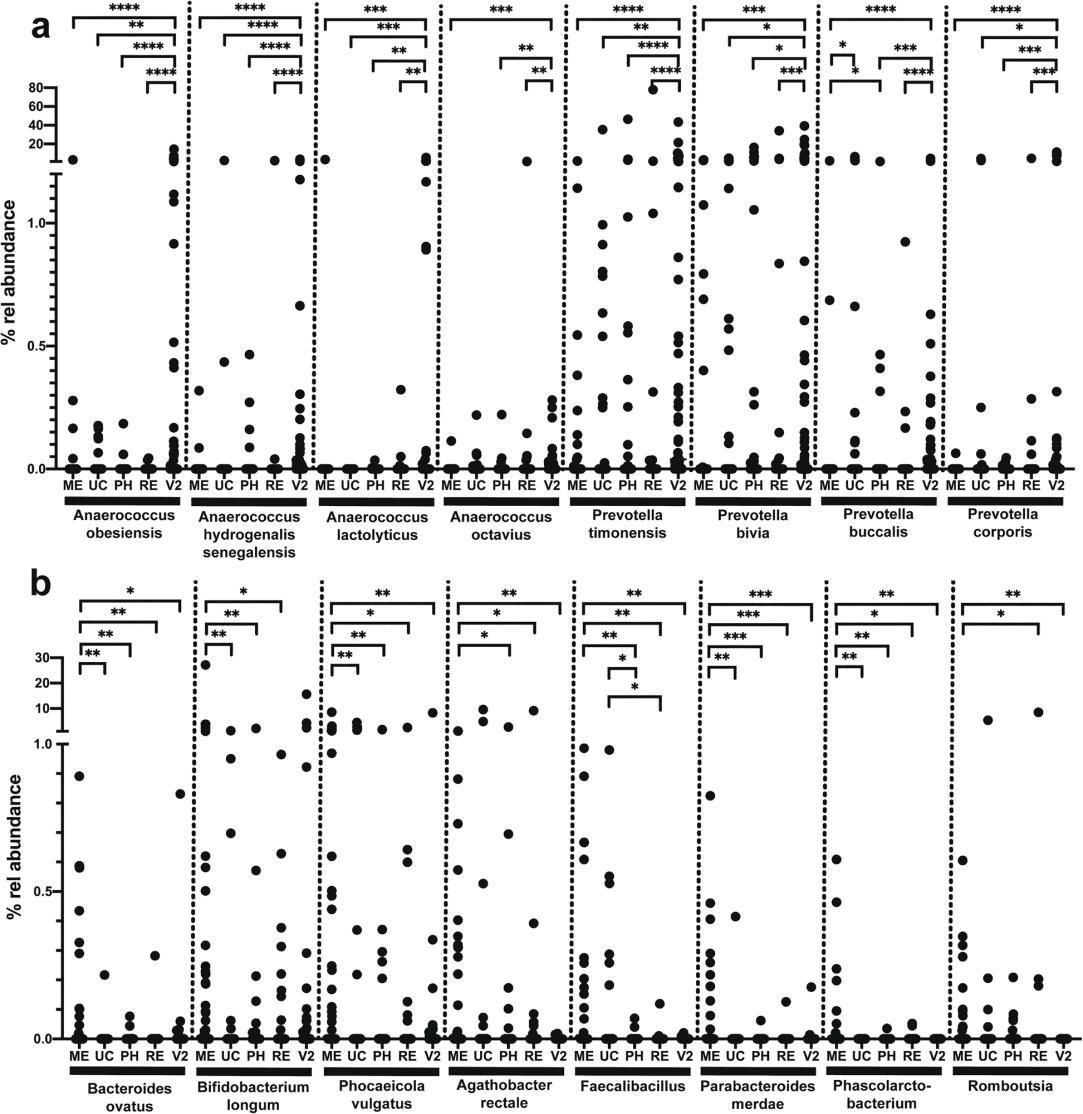
Abundance of selected bacterial taxa which were differentially distributed between meconium (ME), umbilical cord blood (UC), pharyngeal swabs (PH), rectal swabs (RE), and the vaginal microbiota at birth (V2). **a** Taxa of significantly higher abundance in vaginal samples compared to neonatal samples. **b** Taxa of significantly higher abundance in meconium samples compared to other compartments tested. The difference in similarity was analyzed by the Kruskal–Wallis test, and pairwise comparisons were performed by the Dunns’s post hoc test with *p < 0.05, **p < 0.01, ***p < 0.001, and ****p < 0.0001

Among others, different *Anaerococcus* species (*A. hydrogenalis/senegalensis*, *A. lactolyticus*, *A. murdochii*, *A. obesiensis*, and *A. octavius*), *Campylobacter* species (*C. hominis*, *C. ureolyticus*), *Dialister* species (*D. microaerophilus*, *D. propionifaciens*), *Peptoniphilus* species (*P. coxii*, *P. gorbachii*, *P. grossenis*, *P. harei*, and *P. lacrimalis*), or *Prevotella* species (*P. bergensis*, *P. bivia*, *P. buccalis*, *P corporis*, and *P. timonensis*) were significantly higher abundant in vaginal communities compared to the neonatal communities, whereas *C. acnes*, *Acinetobacter junii*, or *Staphylococcus* spp. were less abundant. Meconium samples were characterized by specific taxa enriched in this niche and *Bacteroides ovatus*, *Bifidobacterium longum*, *Parabacteroides merdae*, *Phocaeicola vulgatus*, *Agathobacter rectale*, *Faecalibacillus*, *Phascolarctobacterium*, and *Romboutsia* were of significantly higher abundance in meconium communities compared to others neonatal communities and to maternal vaginal communities, as well (Fig. 7).

The PERMANOVA pairwise analysis between spontaneous vaginal delivery and C-section delivery did not show any significant influence of delivery type on neonatal microbial composition for any of the neonatal sampling groups tested. However, there were also cases where a transfer of vaginal communities to the neonate was clearly observed. As an example, all UC, PH, and RE samples of neonates H06 and R07 were dominated by *U. parvum* as was the mother’s vaginal microbiota at birth and similar observations were made for a possible transfer of *Escherichia/Shigella* in neonate R04 and of *L. crispatus* in neonate H05.

### Prediction of early-onset neonatal sepsis

Sixteen PPROM patients delivered 18 neonates which developed EONS (Additional file 2: Table S2). To evaluate if the vaginal community of the mother could be predictive for EONS, we analyzed if the V0, V1, or V2 communities differed at the species level between mothers giving birth to at least one child suffering from EONS and mothers giving birth to children not suffering from EONS (Table 2).

**Table 2:** Results for pairwise PERMANOVA based on the Bray–Curtis similarities of vaginal communities calculated from standardized relative species abundance data of mothers giving birth to at least one neonate later on suffering from EONS and mothers giving birth to neonates not suffering from EONS

Vaginal sample groups	Pseudo-*F*	*p*
V0	0.42541	0.837
V1	1.2004	0.253
V2	1.8817	0.044*
V2m	1.6819	0.064
V2l	1.7451	0.035*

^*^Significant value

Whereas PERMANOVA did not indicate any difference when comparing V0 and V1 communities (*p* = 0.837 and 0.253, respectively), the difference in the overall community structure was significant when V2 samples were compared (*p* = 0.044). The difference was even more pronounced when only mothers having received antibiotics for an extended period of time were considered (V2l, *p* = 0.035). On the single taxa level, *Anaerococcus obesiensis*, *Anaerococcus lactolyticus*, *Campylobacter ureolyticus*, and *Howardella* trended to be underrepresented in samples of mothers where a child suffers later from EONS, whereas *Escherichia/Shigella*, *Enterococcus faecalis*, *Facklamia*, *Winkia neulii*, *S. aureus*, and *Eremecoccus* trended to be overrepresented in mothers of EONS (Table 3) (Additional file 15: Fig. S5); however, higher sample numbers are necessary to further prove these results.

**Table 3:** Bacterial taxa differentially distributed in the vaginal microbiota of EONS and non-EONS cases

Bacterial taxa	V2		V2m		V2l	
	Mann–Whitney test	Fisher’s exact test	Mann–Whitney test	Fisher’s exact test	Mann–Whitney test	Fisher’s exact test
*Enterococcus faecalis*^a^	0.0081	0.0256	0.0133	0.0098	ns	0.0306
*Escherichia/Shigella*^a^	ns	ns	0.0375	NA	0.0225	ns
*Facklamia*^a^	0.0037	0.0105	0.0014	0.0045	0.0335	ns
*Winkia neuii*^a^	ns	ns	0.0111	0.0314	0.0114	0.0172
*Anaerococcus obesiensis*^b^	0.0394	ns	0.0418	ns	0.0365	ns
*Anaerococcus* lactolyticus^b^	0.0440	ns	ns	ns	ns	ns
*Howardella*^b^	0.0122	0.0135	ns	ns	0.0454	ns
*Staphylococcus aureus*^a^	0.0011	0.0073	0.0064	0.0201	0.0335	ns
*Campylobacter ureolyticus*^b^	0.0336	ns	ns	ns	NA	NA
*Eremecoccus*^a^	NA	NA	0.0064	0.0201	NA	NA
*Uncl. Streptococcus*^a^	ns	ns	0.0363	ns	ns	ns
*Lactobacillus iners*^a^	ns	0.0349	ns	ns	0.0449	0.0306

Uncorrected *p*-values are given

*V2*, all samples collected prior to delivery; *V2M*, vaginal samples from mothers with > 2 days of antibiotic treatment; *V2l*, vaginal samples from mothers with > 6 days of antibiotic treatment; *ns*, not significant (*p* > 0.05); *NA*, taxon present in < 10% of samples

^a^Probable bacterial risk taxa (more abundant in EONs cases)

^b^Probable protective bacterial taxa (more abundant in non-EONs cases)

It was then analyzed if also the neonatal meconium and pharyngeal and rectal swab communities differ between EONS and non-EONS cases. PERMANOVA did not indicate a significant difference between the meconium communities of EONS and non-EONS neonates (*p* = 0.188) whereas significant differences were observed in pharyngeal (*p* = 0.006) and rectal swab (*p* = 0.033) communities. Birth mode had no significant influence on the community structures (meconium, *p* = 0.402; pharyngeal swab, *p* = 0.646; rectal swab, *p* = 0.695).

An analysis of the pharyngeal swab taxa differentially distributed between EONS and non-EONS neonates showed that taxa of increased relative abundance in non-EONS cases are typical skin inhabitants such as *S. epidermidis* or *C. acnes* (Table 4). Whether this increase in the relative abundance of skin organisms is due to an overall lower bacterial load and hence a relatively higher impact of skin-derived bacteria remains to be established. There were no taxa clearly differentially distributed on rectal swabs. In contrast, a higher prevalence of *Bifidobacterium longum* and *Agathobacter rectalis* was indicated in meconium samples, as putative protective organisms (Table 4). As mentioned above, higher sample numbers are necessary to further prove these results.

**Table 4:** Bacterial taxa differentially distributed in the neonatal microbiota of EONS and non-EONS cases

Bacterial taxa	Meconium		Pharyngeal swab	
	Mann–Whitney test	Fisher’s exact test	Mann–Whitney test	Fisher’s exact test
*Bifidobacterium longum*^b^	0.0102	0.0073	ns	ns
*Agathobacter rectale*^b^	0.0489	0.0494	ns	ns
*Staphylococcus epidermidis*^b^	ns	ns	0.0048	ns
*Cutibacterium acnes*^b^	ns	ns	0.0077	ns
*Myxococcales*^b^	0.0259	0.0218	ns	ns
*Sphingomonas*^a^	ns	ns	0.0125	0.0228
*Neisseria perflava/subflava/flavescens*^a^	ns	ns	0.0289	0.0401
*Neisseriaceae*^b^	ns	ns	0.0418	ns

Mann–Whitney test using relative abundance data

Fisher’s exact test using presence/absence data

Uncorrected *p*-values are given

*ns*, not significant (*p* > 0.05)

^a^Probable bacterial risk taxa (more abundant in EONs cases)

^b^Probable protective bacterial taxa (more abundant in EONs cases)

The potential use of vaginal and meconium communities as possible biomarkers to discriminate and predict EONS and non-EONS cases was tested using three different modeling techniques (logistic regression (LR), linear support vector machine (SVM), and random forest classifier (RF)). Best performances were achieved with logistic regression modeling for vaginal and pharyngeal swab communities, and with support vector machine modeling for meconium communities using cube root transformed data.

**Fig. 8:**
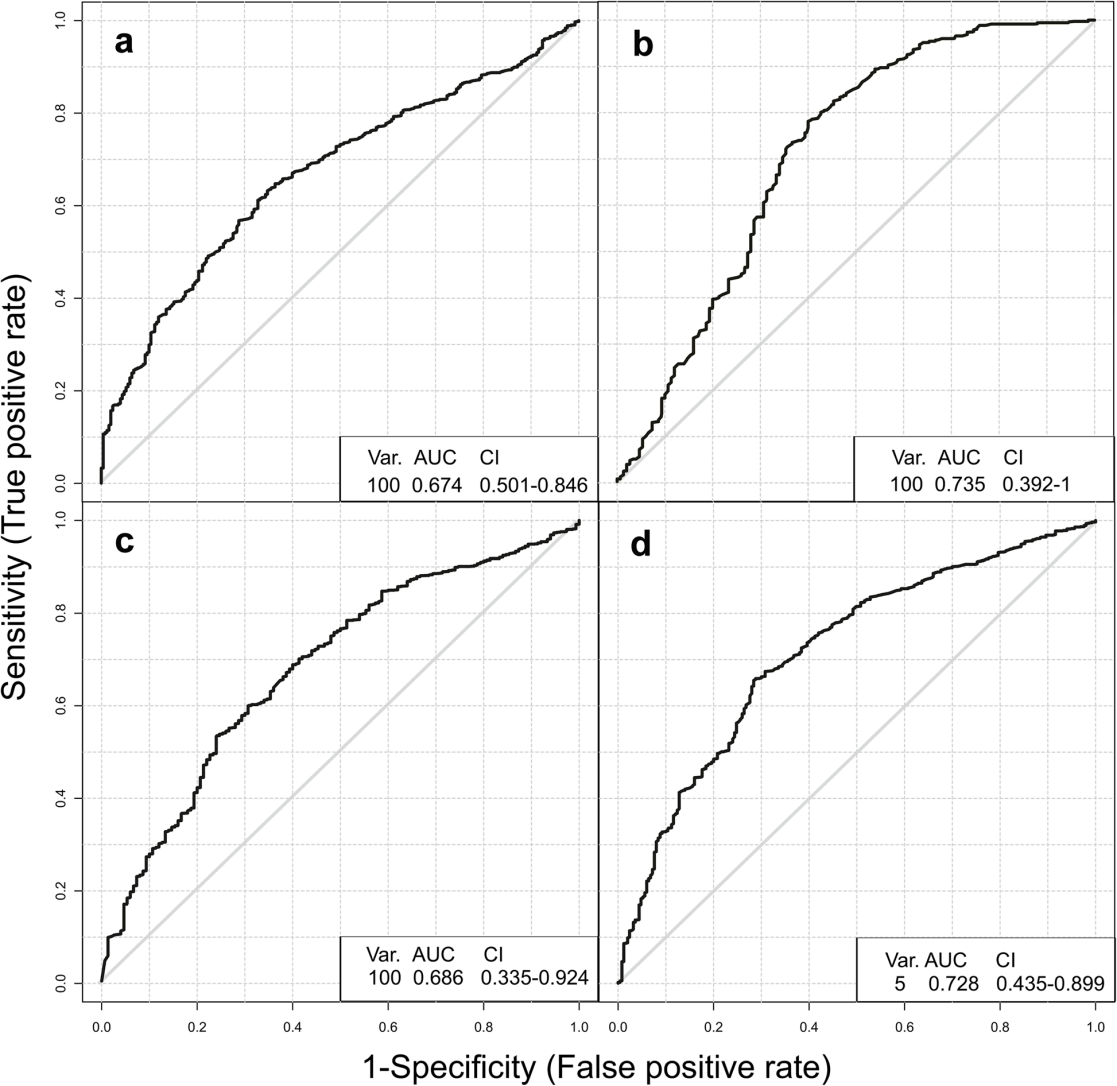
Using microbial communities for the prediction of EONS. ROC curves for a logistic regression (**a**,** b**, **d**) and a support vector machine classifier (**d**) trained on 5–100 taxa of vaginal (**a** all V2 samples and **b** V2l samples > 6 days antibiotic treatment), meconium (**c**), and pharyngeal swab communities (**d**). The amount of taxa used (Var), the area under the curve (AUC), and the confidence interval (CI) are given as inserts

Areas under the receiver operating characteristic curves (ROC-AUC) of up to 0.675, 0.736, 0.672, and 0.685 could be achieved for V2 communities (all samples), V2l communities (vaginal communities under antibiotic treatment for > 6 days), meconium communities, and pharyngeal swab communities, respectively (Fig. 8). The 5-feature pharyngeal model was capable to correctly classify 86% of EONS cases. However, only 58% of non-EONs cases were correctly classified. Here, the best performance was achieved using the 100 features meconium model, which could correctly classify 78% of EONS and 84% of non-EONS cases. Also, the vaginal communities showed a reasonable predictive power for classifying EONS cases, with a 64% true positive and a 69% true negative rate. Communities having been subject to extended antibiotic treatment showed a slightly enhanced predictive power (67% true positive and a 77% true negative rate) (Additional file 16: Fig. S6).

Then, three- to four-taxon models were evaluated on a training cohort consisting exclusively of samples from the two trial sites Halle and Rostock hospitals. Models were validated using an independent cohort of samples from the third trial site Jena hospital. For all V2 samples, the model comprising *Escherichia/Shigella* and *Facklamia* (risk taxa) as well as *Anaerococcus obesiensis* and *Campylobacter ureolyticus* (protective taxa) yielded AUCs of 0.674 and 0.788, respectively. Only a few samples were available to perform this analysis for V2l samples. However, also here, high AUC values of 0.975 and 0.79 were obtained using the risk taxa *Escherichia/Shigella* and *Winkia neuii* and the protective taxon *Anaerococcus obesiensis* as features. Finally, EONs cases could also be predicted at a reasonable rate from neonatal meconium communities with the protective taxa *Bifidobacterium longum*, *Agathobacter rectale*, and *S. epidermidis* as features yielding AUCs of 0.592 and 0.753 for training cohort and validation cohort, respectively, while AUCs of 0.706 and 0.664 for prediction of EONS by the pharyngeal community protective taxa *S. epidermidis*, *C. acnes*, and Neisseriales were observed (Additional file 17: Fig. S7).

## Discussion

Preterm premature rupture of the fetal membranes (PPROM) precedes one-third of all spontaneous preterm births. Vaginal bacterial communities depleted in *Lactobacillus* species and high in diversity have been reported as a risk factor for subsequent PPROM [36]. In the present study, only roughly two-thirds of the pregnant women harbored a vaginal microbiota dominated by *Lactobacillus* species at the time of hospital admission, in accordance with previous reports on women suffering from PPROM [4, 36]. Typical vaginal pathogens such as *S. agalactiae*, *Fannyhessea vaginalis*, *G. vaginalis*, or *U. parvum/urealyticus* were frequently observed. As the uterine cavity, placenta, and fetus are subsequently exposed to these bacteria, the risk of chorioamnionitis, funisitis, and colonization of the neonate increases. Thus, antibiotic treatment was recommended [38], also based on a clinical trial where antibiotic treatment had been shown to be associated with the prolongation of pregnancy, and fewer positive blood cultures [39]. However, it was assumed as unlikely that for example treatment with erythromycin prevents ascending infections, as erythromycin concentrations in the vagina may reach levels effective against *Lactobacillus* species but not against most vaginal pathogens [3, 40]. A recent cohort study of mother-infant couples who delivered after PPROM showed an increased incidence of antimicrobial-resistant Gram-negative organisms on placental swabs and all cases of neonatal early-onset sepsis occurred in those who received erythromycin [41]. Based on a clinical trial performed by Mercer and colleagues, ampicillin and amoxicillin together with erythromycin are widely used, with erythromycin being substituted by azithromycin due to fewer side effects [42] and a recent study [43] showed that women under such therapy had a longer median latency from time of rupture of membranes to delivery than women prescribed erythromycin. However, the microbiota analysis performed here shows, in agreement with previous reports [4], that antibiotic treatment resulted in an increase in the vaginal microbial diversity and *Lactobacillus* was eliminated from vaginal communities previously dominated by species of that genus. In contrast, various communities became dominated by *U. parvum* with possible negative consequences [44]. The commonly used single 1 g maternal azithromycin dose may not be optimal to maintain sufficient antibiotic concentrations for the expected 7-day course in the setting of PPROM [45]. Interestingly, only 4 of 29 pregnancies where antibiotic treatment had occurred for < 48 h resulted in an EONS case after delivery (14%) whereas 10 of 34 (29%) extensively treated pregnancies gave rise to EONS cases. Even though this difference is not statistically significant, it is addressing the central problem of pregnancy prolongation after PPROM weighting the benefits of maturing and the potential risk of exposition time to ascending bacterial contamination of the amniotic cavity. It should also be noted that upon the termination of antibiotic treatment, thirteen out of fourteen communities remained dysbiotic and only one community recovered to the initial *L. crispatus* dominance. Clearly, alternative methods to antibiotic treatment need to be evaluated such as the simultaneous use of probiotics [46] or individualized management including amniotic fluid and vaginal microbiota analysis and targeted treatment [47].

We had indicated above that *Anaerococcus obesiensis*, *Anaerococcus lactolyticus*, *Campylobacter ureolyticus*, and *Howardella* trended to be underrepresented in samples of mothers where a child suffers later from EONS, whereas *Escherichia/Shigella*, *Enterococcus faecalis*, *Facklamia*, *Winkia neuii*, *S. aureus*, and *Eremococcus* trended to be overrepresented. There had been no significant difference in the abundance and prevalence of *Ureaplasma* between mothers where the child suffered later from EONS and those who did not, even though a case of *Ureaplasma* caused EONS and severe bronchopulmonary dysplasia of the preterm infant was documented [44]. In fact, *Ureaplasma* species, the most common microbes found in amniotic fluid and placenta after preterm birth, have previously been correlated with chorioamnionitis and preterm labor [48], and especially in the context of PPROM they have to be considered as a harmful pathogen for the preterm infant. Typical pathogens involved in chorioamnionitis, besides *Ureaplasma* and *Gardnerella*, comprise *Fusobacteria* and specially *E. coli* [49] in accordance with *E. coli* as a potential risk factor for EONS when detected in the vaginal microbiota before delivery. The organisms most frequently involved in EONS are *Streptococcus agalactiae* and *E. coli*, with additional pathogens comprising *Staphylococcus aureus*, *Enterococcus* spp., and *Haemophilus influenzae* among others [5] in accordance with the identification of *E. faecalis* and *S. aureus* as further potential risk factors for EONS when present in the vaginal microbiota.

Already two decades ago, *W. neuii* has been characterized as a pathogen causing not only chorioamnionitis but also neonatal sepsis [50]. Since then, this species has been identified as a cause of chorioamnionitis and neonatal sepsis also in other cases [51, 52]. *W. neuii* (and *E. faecalis*) were also shown to enhance *G. vaginalis* virulence in bacterial vaginosis [53] and may be a more important pathogen than previously thought.

*Facklamia* strains (obviously often misidentified as viridans group *Streptococcus*) are reported as emerging pathogens and were isolated from human infections, including sepsis, genitourinary infections, or wound infections [54]. *Facklamia hominis* has been described as a cause of chorioamnionitis and maternal sepsis and may have been responsible for an episode of sepsis in the neonate [55]. Importantly, resistance against erythromycin and other antibiotics seems to be spread among *Facklamia* isolates [54–56], and their importance for chorioamnionitis and neonatal sepsis clearly needs to be further evaluated.

It is well documented that the extensive colonization of the human gut begins postpartum [57]. Moreover, it was generally assumed that the neonate is born sterile, and only after delivery the neonate is populated by bacteria [58], which would mean that the meconium is sterile in utero but rapidly colonized after birth [59]. However, various publications have shown that meconium is not sterile [60]. Moreover, the meconium microbiota was shown to share more features with the amniotic fluid microbiota than the maternal fecal and vaginal microbiota suggesting that the amniotic fluid microbiota contributed significantly to the seeding of the meconium microbiota [23]. Given that the fetus swallows amniotic fluid throughout the second and third trimesters, such a sharing of a large portion of the microbiota is expected [21]. Considering that the cohort analyzed here consisted exclusively of PPROM patients, where ruptured membranes offer the possibility of ascending bacterial colonization from the maternal vagina, it is also probable that in various of them a colonization of the amniotic fluid had occurred. Accordingly, typical pathogens of chorioamnionitis such as *Ureaplasma* [49] were highly abundant in various meconium samples analyzed here, and one sample, where the neonate later suffered from EONS, was dominated by *Fusobacterium nucleatum*, previously described as responsible for various adverse pregnancy outcomes and even neonatal sepsis [61]. However, besides these organisms, meconium commonly contains Gammaproteobacteria such as *E. coli* and Bacilli such as *Enterococcus*, *Staphylococcus*, and *Streptococcus* but also *Bifidobacterium* and *Phocaeicola vulgatus* [59, 62], organisms also identified here as major members of the meconium microbiota. Importantly, the microbiota of the meconium was not influenced to a significant extent by the mode of delivery, and comparisons of the gut microbiota at delivery revealed that in both Cesarean and vaginally delivered infants, *Bifidobacterium* and *Bacteroides/Phocaeicola* were appreciably present [60]. Notably, meconium samples harbored bacteria predicted to originate from the maternal stool or oral cavity. Thus, meconium communities may originate from a vertical ascension from the vagina and a hematogenous route through the placenta after translocation from the digestive tract [63, 64] and postpartum from environmental sources.

It was already early suggested that the type of bacteria detected in meconium may influence childhood health [65], and as an example, an association between neonatal jaundice and the meconium microbiome was observed [66] with a higher abundance of *Bifidobacterium pseudolongum* associated with a lower risk. Colonization with *Bifidobacterium* early in life has also been associated with protection from necrotizing enterocolitis [67] and late-onset neonatal sepsis [68]. In fact, the early colonization with *Bifidobacterium* may be crucial for human health status. They are important for shaping the immune system and interact with the host through cell surface-associated exopolysaccharides, fimbriae or pili, and secreted serine protease inhibitors, but they are also important metabolically through the degradation of diet-derived glycans and host-provided carbohydrates [63]. It is, thus, not astonishing that *Bifidobacterium*, here *B. longum*, could be identified as putatively protective against EONS. It might be speculated, however, that Bifidobacteria are not protective per se but that they are indicative for a more mature gut. In fact, the gestational age of neonates the meconium of which harbored *B. longum* was slightly higher compared to that of neonates where no *B. longum* was detected (32.4 ± 2.0 versus 30.9 ± 3.2 weeks, *p* = 0.028) and further studies with larger cohorts are necessary to define better the effect of *B. longum*. *A. rectale* was indicated as a second organism with a probably protective effect. In contrast to *B. longum*, there was no difference in gestational age of neonates harboring *A. rectale* and those that do not harbor this organism in their meconium (32.0 ± 2.04 versus 31.4 ± 3.0 weeks, *p* = 0.377).

## Conclusions

The vaginal microbiota analysis reveals complex bacterial communities in PPROM patients, and the analysis is capable of differentiating between *Lactobacillus* species. It can identify bacterial genera and species such as *Facklamia* spp. or *Winkia neuii*, which are not captured in conventional diagnostics but may be relevant for PPROM and/or EONS (or neonatal complications) and therefore relevant for adequate risk assessment and individual therapy.

The standard antibiotic treatment applied to PPROM patients contributes to the loss of potentially protective *Lactobacillus* species in EONS after PPROM leading to a rise in community diversity and persistence of potential pathogens and alternative methods to antibiotic treatment need to be evaluated.

The neonatal microbiota differs in composition by sampling site between the pharynx/rectum (surface) and meconium (processed amniotic fluid). Identification of potentially beneficial or harmful species in this specimen, where culture-based conventional diagnostics are often limited, may help to identify individual treatment strategies in preterm neonates after PPROM and an individual risk assessment.

Biomarkers, which have the potential to be used to predict EONS, could be identified from both the vaginal microbiota at birth as well as from the meconium. Microbiota analysis may therefore be a valuable diagnostic tool in the risk assessment of EONS after PPROM and has to be further evaluated in clinical settings.

## Supplementary Information

**Additional file 1: Table S1.** Antibiotics given to mothers.

**Additional file 2: Table S2. **PPROM patients and neonates included in the current study.

**Additional file 3: Table S3. **Classification of vaginal samples taken at delivery.

**Additional file 4: Table S4.** Nucleotide sequences, annotation and sequence count data of all sequence variants determined using Illumina-based amplicon deep-sequencing across 389 samples.

**Additional file 5: Table S5.** Nucleotide sequences, annotation and relative abundance data of sequence variants determined using Illumina-based amplicon deep-sequencing across kit control samples.

**Additional file 6: Table S6. **Relative abundance data of all sequence variants determined using Illumina-based amplicon deep-sequencing across those 378 samples with >2000 read counts.

**Additional file 7: Table S7.** Relative abundance data of all species level taxa across 166 vaginal swab samples.

**Additional file 8: Fig. S1.** Changes in microbial community structure upon antibiotic treatment. The treatment time is indicated as well as the Bray Curtis similarity (in %) of the community structure at time of admission to the hospital. The similarity trend is indicated by a line following a one phase decay.

**Additional file 9: Fig. S2.** Microbial composition of vaginal samples of PPROM patients before delivery (V2). Heatmap includes taxa with a relative abundance > 10% in at least one sample. Samples where a single taxon shows a relative abundance > 50% of relative abundance are indicated by a specific color code (dominance group). Hierarchical cluster based on Bray-Curtis similarity analysis of vaginal bacterial communities of V2 samples (*n*=63).

**Additional file 10: Fig. S3.** Differences in global bacterial community structure of vaginal samples as assessed by non-metric multidimensional scaling (nMDS). The global community structures are based on standardized species abundance data and shown for all patients where sampling at three different time points had been performed. **a**, patients treated with antibiotics until delivery; **b**, patients treated with antibiotics no longer than 2 days before delivery. Patients are indicated by different color codes and symbols. 0; samples taken at hospital admission; 1; samples taken after 2-6 days of antibiotic treatment; 2; samples taken later than 6 days after hospital admission.

**Additional file 11: Table S8.** Relative abundance data of species level taxa across 212 neonatal samples.

**Additional file 12: Fig. S4.** Microbial composition of vaginal and meconium samples. Heatmap includes taxa with a relative abundance > 10% in at least one sample. Samples where a single taxon shows a relative abundance > 50% of relative abundance are indicated by a specific color code (dominance group). Hierarchical cluster based on Bray-Curtis dissimilarity analysis of vaginal bacterial communities of V2 and ME samples (*n*=103).

**Additional file 13: Table S9.** Pairwise PERMANOVA based on the Bray-Curtis similarities of vaginal and neonatal communities calculated from standardized relative species abundance data.

**Additional file 14: Table S10.** Taxa differentially distributed between the vaginal microbiota at time of delivery and neonatal microbiota. The difference in distribution was tested by the Kruskal-Wallis test with Benjamini-Hochberg corrections for multiple comparisons. Groups of samples were considered significantly different if the adjusted p-value was <0.05. Only taxa fulfilling this criterion are shown. Pairwise comparisons were performed between vaginal samples taken at birth (V2), meconium (ME), umbilical cord blood (UC), pharyngeal swab (PH) and rectal swab (RE) samples. p-Values were corrected by the Dunn`s test.

**Additional file 15: Fig. S5.** Differences in vaginal global bacterial community structures based on standardized species abundance data. Non-metric multidimensional scaling (nMDS) plot with superimposed bubbles representing the relative abundance (in %) of (a) *Escherichia/Shigella *or (b) *Anaerococcus obesiensis. *Red color shows EONS (E), and gray/black color shows non-EONS (N) cases.

**Additional file 16: Fig. S6. **Confusion matrices summarizing the performance of models for the prediction of EONS. Each row of the confusion matrix shows the number of samples in an actual class while each column shows the number of samples in a predicted class. Tiles showing the number of correctly classified cases are colored magenta (EONS) or green (non EONS) a, confusion matrix based on 5 V2 taxa; b, confusion matrix based on 5 V2L taxa; c, confusion matrix based on 100 meconium taxa; d, confusion matrix based on a 5 pharyngeal taxa.

**Additional file 17: Fig. S7. **Identification of potential biomarkers for microbial prediction of EONS. ROC curves showing 3-4 taxa logistic regression support vector machine SVM classifier performance in a training (CV) and an independent validation cohort (holdout). a, all V2 samples; b, V2L samples >6 days antibiotic treatment; c, meconium; d, pharyngeal swab community (D).

## Data Availability

Sequence data are deposited in the Sequence Read Archive under accession number PRJNA804385 (https://www.ncbi.nlm.nih.gov/bioproject/PRJNA804385/) [69].

## Abbreviations

ASV: Amplicon sequence variants
EONS: Early onset neonatal sepsis
LONS: Late-onset neonatal sepsis
LR: Logistic regression
ME: Meconium
nMDS: Non-metric multidimensional scaling
PERMANOVA: Permutational multivariate analysis of variance
PH: Pharyngeal swab
PPROM: Preterm premature rupture of the membranes
RDP: Ribosomal database project
RE: Rectal swab
RF: Random forest classifier
ROC-AUC: Area under the receiver operating characteristic curve
SVM: Linear support vector machine
UC: Umbilical cord blood
