# Supplementary figures and images for: Vaginal and neonatal microbiota in pregnant women with preterm premature rupture of membranes and consecutive early onset neonatal sepsis
Source: BMC Med. 2023 Mar 13;21:92. doi: 10.1186/s12916-023-02805-x (PMC10009945; doi:10.1186/s12916-023-02805-x)

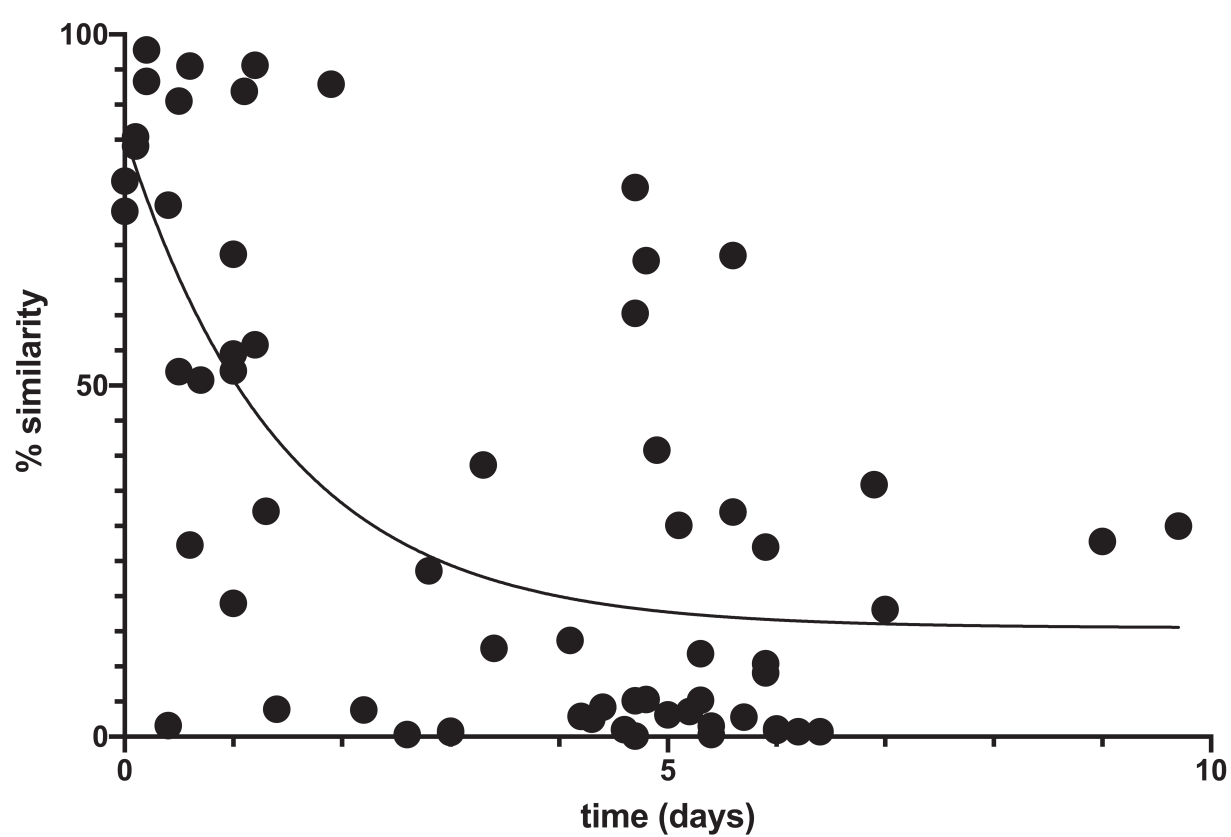

Supplement: Supplementary file 8 — Additional file 8: Fig. S1. Changes in microbial community structure upon antibiotic treatment. The treatment time is indicated as well as the Bray Curtis similarity (in %) of the community structure at time of admission to the hospital. The similarity trend is indicated by a line following a one phase decay. [file 12916_2023_2805_MOESM8_ESM.pdf]

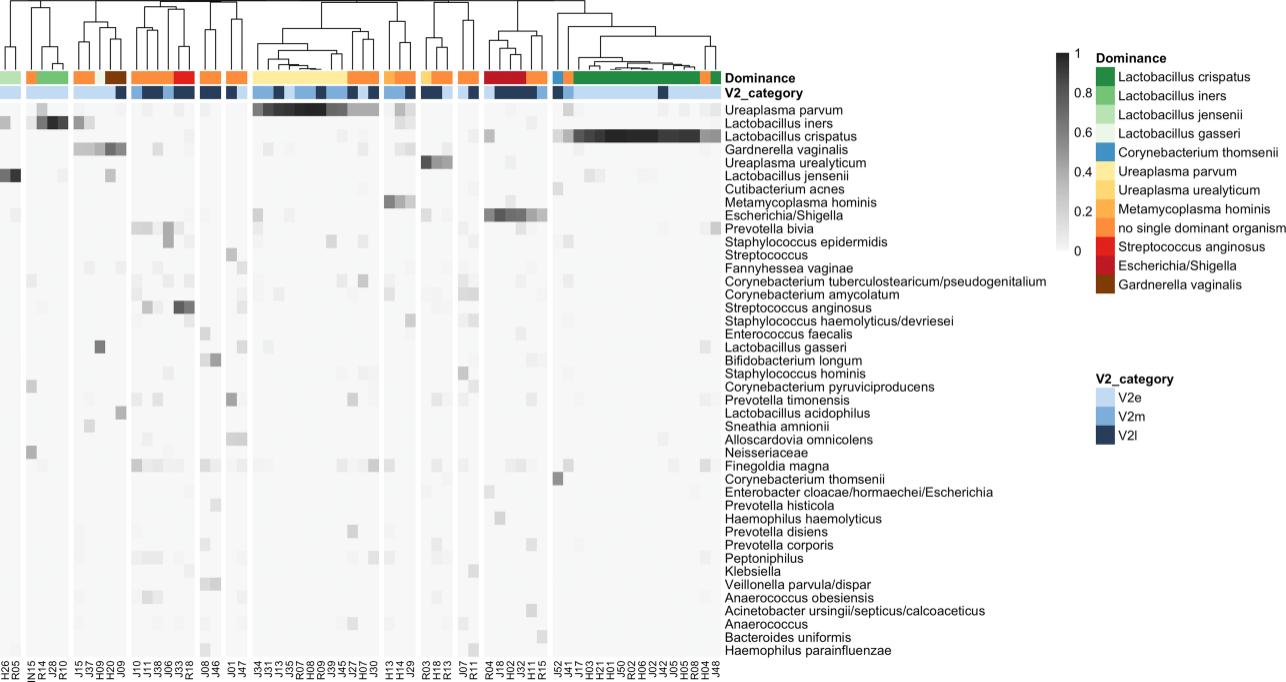

Supplement: Supplementary file 9 — Additional file 9: Fig. S2. Microbial composition of vaginal samples of PPROM patients before delivery (V2). Heatmap includes taxa with a relative abundance > 10% in at least one sample. Samples where a single taxon shows a relative abundance > 50% of relative abundance are indicated by a specific color code (dominance group). Hierarchical cluster based on Bray-Curtis similarity analysis of vaginal bacterial communities of V2 samples (n=63). [file 12916_2023_2805_MOESM9_ESM.pdf]

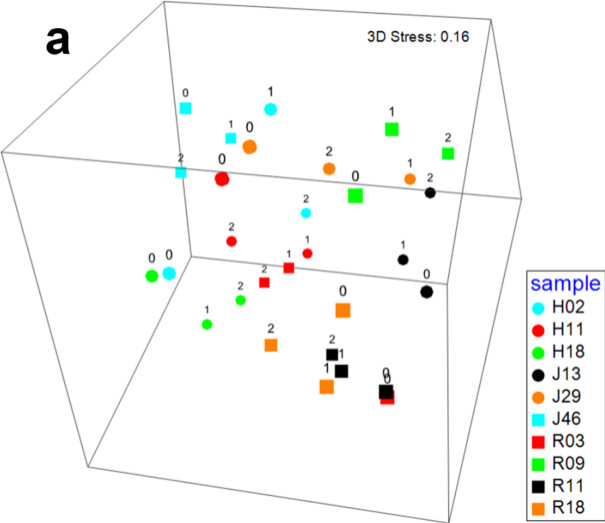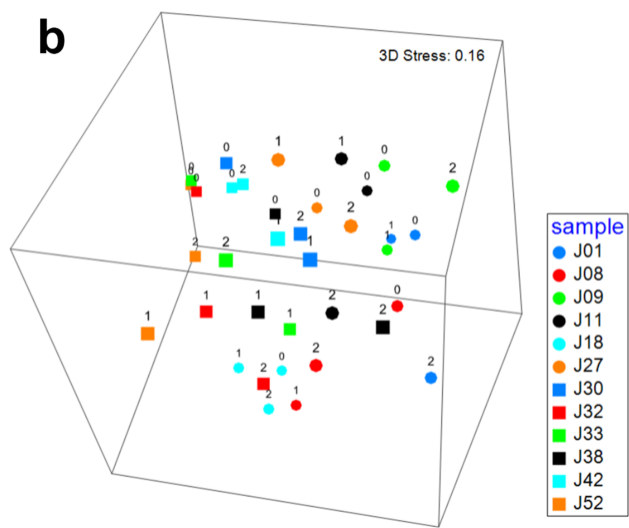

Supplement: Supplementary file 10 — Additional file 10: Fig. S3. Differences in global bacterial community structure of vaginal samples as assessed by non-metric multidimensional scaling (nMDS). The global community structures are based on standardized species abundance data and shown for all patients where sampling at three different time points had been performed. a, patients treated with antibiotics until delivery; b, patients treated with antibiotics no longer than 2 days before delivery. Patients are indicated by different color codes and symbols. 0; samples taken at hospital admission; 1; samples taken after 2-6 days of antibiotic treatment; 2; samples taken later than 6 days after hospital admission. [file 12916_2023_2805_MOESM10_ESM.pdf]

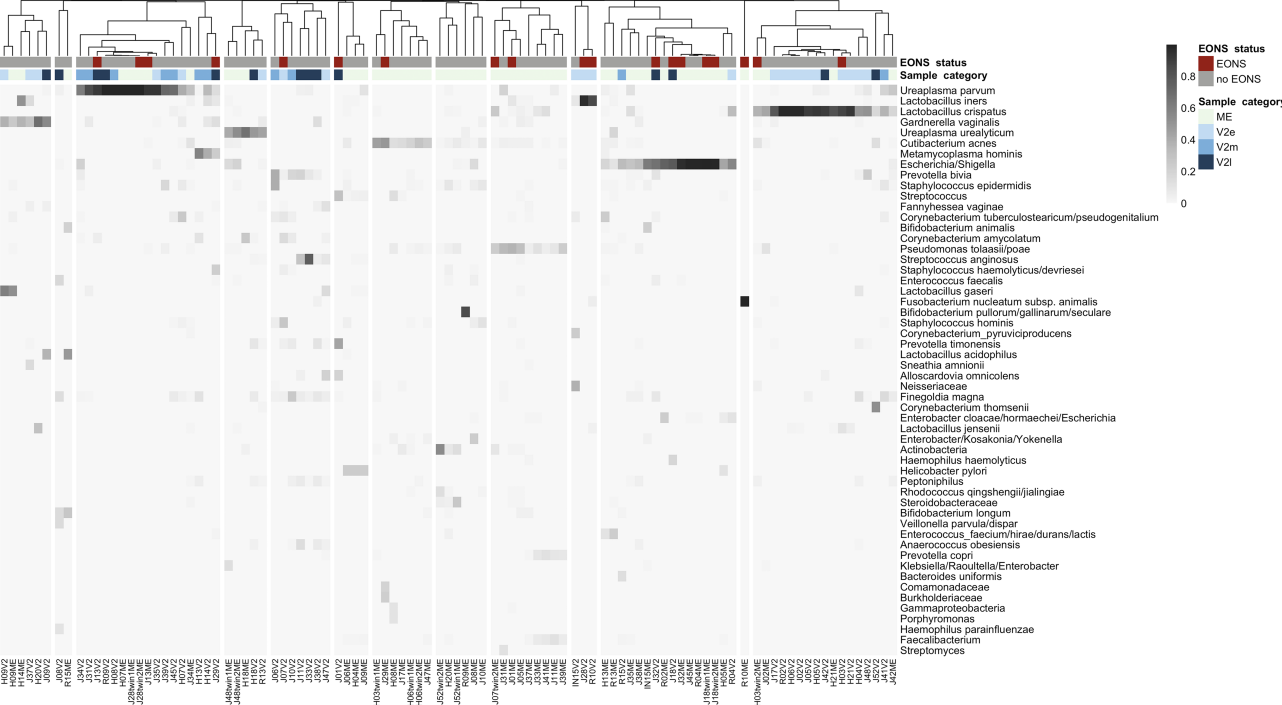

Supplement: Supplementary file 12 — Additional file 12: Fig. S4. Microbial composition of vaginal and meconium samples. Heatmap includes taxa with a relative abundance > 10% in at least one sample. Samples where a single taxon shows a relative abundance > 50% of relative abundance are indicated by a specific color code (dominance group). Hierarchical cluster based on Bray-Curtis dissimilarity analysis of vaginal bacterial communities of V2 and ME samples (n=103). [file 12916_2023_2805_MOESM12_ESM.pdf]

Escherichia\_Shigella

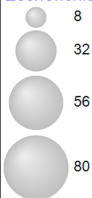

**a**

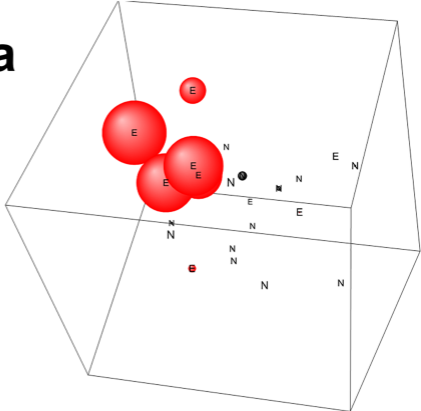

Anaerococcus\_obesiensis

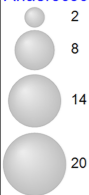

**b**

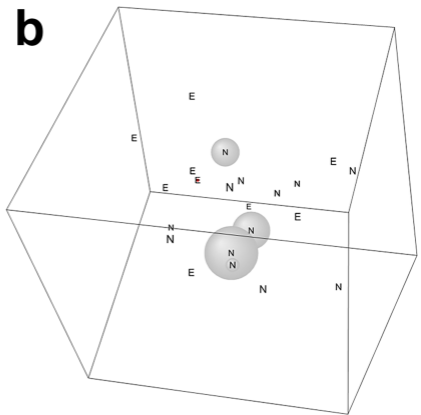

Supplement: Supplementary file 15 — Additional file 15: Fig. S5. Differences in vaginal global bacterial community structures based on standardized species abundance data. Non-metric multidimensional scaling (nMDS) plot with superimposed bubbles representing the relative abundance (in %) of (a) Escherichia/Shigella or (b) Anaerococcus obesiensis. Red color shows EONS (E), and gray/black color shows non-EONS (N) cases. [file 12916_2023_2805_MOESM15_ESM.pdf]

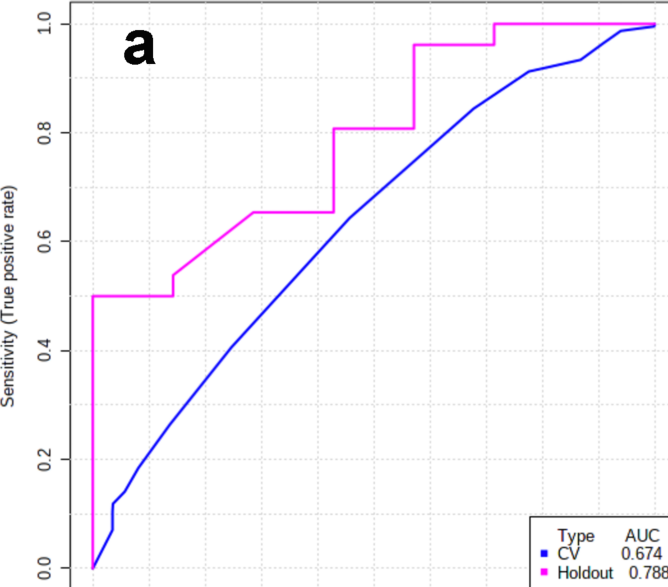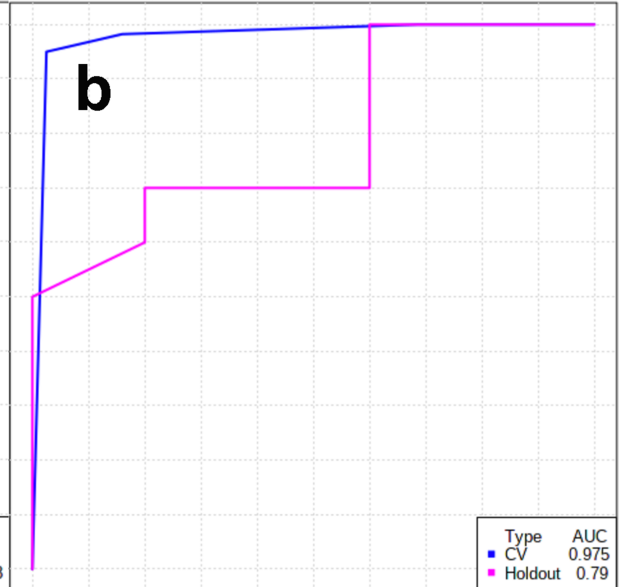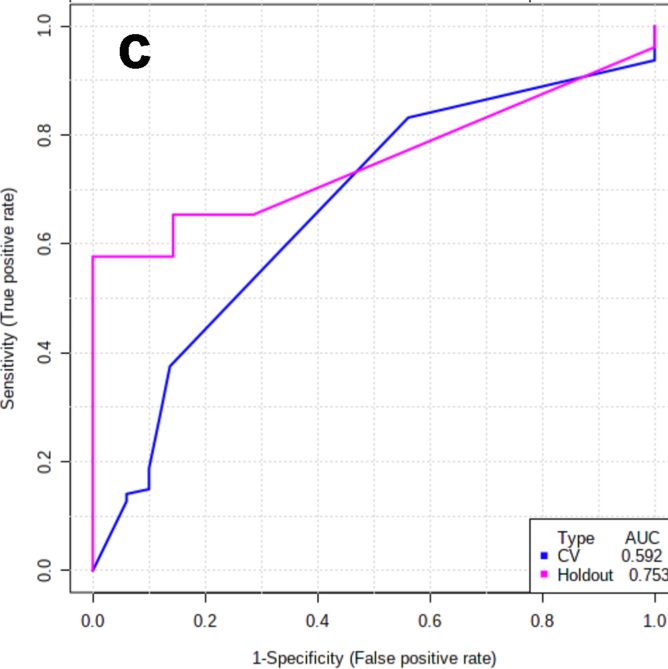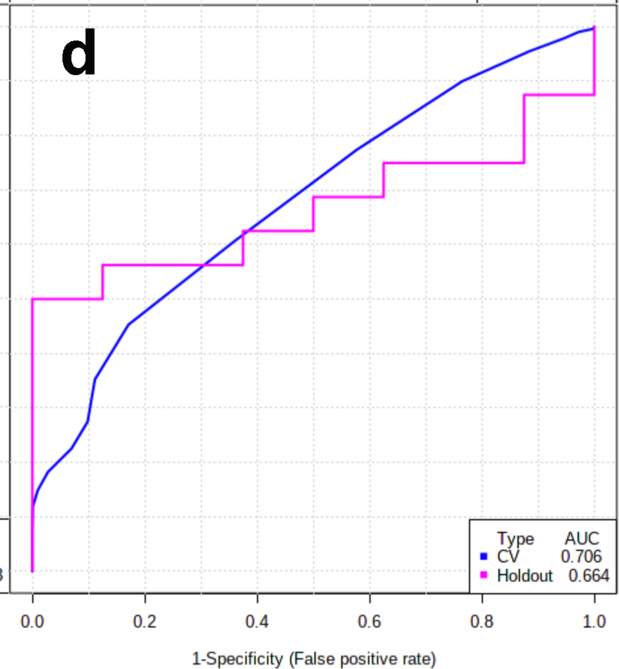

Supplement: Supplementary file 17 — Additional file 17: Fig. S7. Identification of potential biomarkers for microbial prediction of EONS. ROC curves showing 3-4 taxa logistic regression support vector machine SVM classifier performance in a training (CV) and an independent validation cohort (holdout). a, all V2 samples; b, V2L samples >6 days antibiotic treatment; c, meconium; d, pharyngeal swab community (D). [file 12916_2023_2805_MOESM17_ESM.pdf]
