# Supplementary material for: Vaginal and neonatal microbiota in pregnant women with preterm premature rupture of membranes and consecutive early onset neonatal sepsis
Source: BMC Med. 2023 Mar 13;21:92. doi: 10.1186/s12916-023-02805-x (PMC10009945; doi:10.1186/s12916-023-02805-x)
Supplement: Supplementary file 16 — Additional file 16: Fig. S6. Confusion matrices summarizing the performance of models for the prediction of EONS. Each row of the confusion matrix shows the number of samples in an actual class while each column shows the number of samples in a predicted class. Tiles showing the number of correctly classified cases are colored magenta (EONS) or green (non EONS) a, confusion matrix based on 5 V2 taxa; b, confusion matrix based on 5 V2L taxa; c, confusion matrix based on 100 meconium taxa; d, confusion matrix based on a 5 pharyngeal taxa. [file 12916_2023_2805_MOESM16_ESM.pdf]

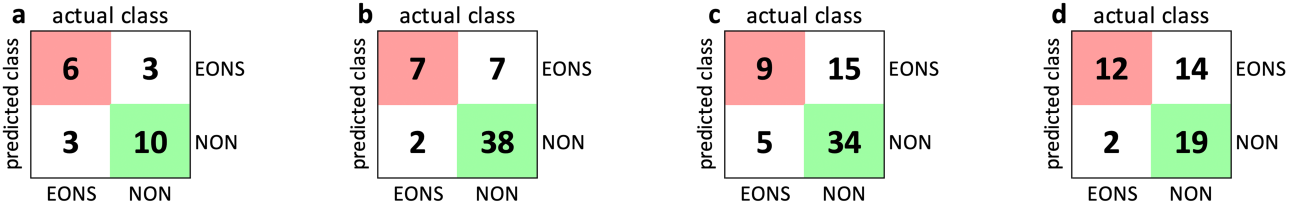

a
actual class
predicted class
6
3
EONS
3
10
NON
EONS
NON
b
actual class
predicted class
7
7
EONS
2
38
NON
EONS
NON
c
actual class
predicted class
9
15
EONS
5
34
NON
EONS
NON
d
actual class
predicted class
12
14
EONS
2
19
NON
EONS
NON
